# Perivascular SPP1 Drives Microglial Synaptic Engulfment After Ischemic Stroke

**DOI:** 10.1002/cns.70886

**Published:** 2026-04-28

**Authors:** Chenchen Xu, Xiaoxiao Li, Nan Cheng, Rui Zhao, Xin Wang, Wenxin Xia, Jianjian Dong, Yongsheng Han

**Affiliations:** ^1^ Institute of Neurology Anhui University of Chinese Medicine Hefei China; ^2^ Wannan Medical College Wuhu China; ^3^ The Affiliated Hospital of Institute of Neurology Anhui University of Chinese Medicine Hefei China

**Keywords:** barrier integrity, glial‐vascular unit, ischemic stroke, microglia synaptic engulfment, perivascular signaling

## Abstract

**Objective:**

Following ischemic stroke (IS), activated microglia activity could contribute to neuronal injury and blood–brain barrier (BBB) disruption. The upstream vascular‐derived signal initiating this transition remains unclear; therefore, we investigated whether perivascular SPP1 regulates microglia‐mediated synapse engulfment during IS.

**Methods:**

Male C57BL/6 mice were assigned to sham, sh*Spp1*, middle cerebral artery occlusion/reperfusion (MCAO/R), and MCAO/*R* + sh*Spp1* groups. Cerebral perfusion was assessed using laser speckle contrast imaging and super‐resolution vascular imaging, while neuronal injury was evaluated using Nissl and TUNEL staining. Proteomic profiling of the ischemic penumbra identified regulators of microglia‐mediated synaptic remodeling. Synaptic structure and glial‐vascular unit (GVU) integrity were examined using transmission electron microscopy, immunofluorescence, and molecular analyses. Behavioral outcomes were assessed using the open‐field, Barnes maze, rotarod, and wire‐hanging tests.

**Results:**

Compared with sham controls, MCAO/R mice displayed increased microglial synaptic engulfment and ultrastructural synaptic damage in the ischemic penumbra, accompanied by reduced synaptic protein expression. Proteomic analysis revealed upregulation of inflammatory and vascular‐related pathways, with marked upregulation of SPP derived from perivascular macrophages. *Spp1* silencing attenuated neuroinflammation, reduced infarct volume, improved cerebral perfusion, preserved GVU integrity, and alleviated behavioral deficits. *Spp1* suppression also reduced microglial synaptic engulfment in vivo and restored synaptic protein and mRNA levels in vitro.

**Conclusion:**

Targeting perivascular SPP1 suppresses excessive microglia‐mediated synaptic engulfment, preserves BBB integrity and synaptic architecture, and offers a GVU‐centered therapeutic strategy for IS.

## Graphical abstract

BBB damage post ischemic stroke triggers perivascular SPP1 release, which drives microglia‐mediated synapse engulfment in the ischemic penumbra of the MCAO/R mouse model.

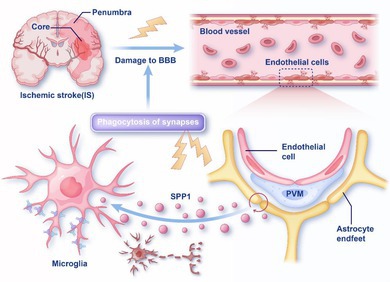

## 1 Introduction

Ischemic stroke (IS) remains a major global health issue, accounting for a significant proportion of long‐term disability and mortality worldwide [1, 2]. The pathophysiology of IS begins with the occlusion of a cerebral artery, disrupting blood flow and creating an area of irreversible tissue damage, or the ischemic core, surrounded by a region of salvageable tissue known as the ischemic penumbra [3]. While neuronal death occurs rapidly in the ischemic core, a secondary wave of damage unfolds in the penumbra, characterized by progressive synapse loss [4, 5, 6, 7]. This continued synaptic degradation significantly contributes to the long‐term neurological deficits observed in stroke survivors [8].

Microglia, the resident immune cells of the central nervous system (CNS), play a central role in the brain's response to ischemic injury. Under normal conditions, microglia help maintain synaptic plasticity by pruning excess or weak synapses, a process crucial for brain development and circuit refinement [9, 10]. However, following ischemic injury, microglia become activated, and their phagocytic activity is dramatically upregulated. This heightened response serves a dual function. First, microglial phagocytosis is essential for clearing debris and limiting inflammation; second, dysregulated activation leads to the excessive engulfment of stressed, yet potentially viable, synapses in the penumbra [11, 12]. This pathological synapse removal disrupts surviving neural networks, exacerbates neuronal dysfunction, and contributes to cognitive and motor impairments [13, 14, 15]. Notably, similar phenomena have been observed in other neurodegenerative conditions, such as Alzheimer's disease (AD) [16].

The integrity of the brain parenchyma is maintained by the glial‐vascular unit (GVU), a complex structure composed of neurons, glial cells (astrocytes, microglia, and oligodendrocytes), and vascular components (endothelial cells and pericytes). The GVU is crucial for regulating blood–brain barrier (BBB) integrity, cerebral blood flow, and neuroinflammation. IS profoundly disrupts GVU homeostasis, resulting in BBB breakdown, inflammatory cell infiltration, and exacerbation of neuronal injury [17]. Microglia, as a key component of the GVU, play pivotal roles in mediating these post‐stroke outcomes [18, 19, 20]. However, the molecular signals driving excessive microglial synapse engulfment and GVU disruption after IS remain poorly understood, limiting the development of effective therapeutic strategies.

Secreted phosphoprotein 1 (SPP1), also known as osteopontin, is a multifunctional glycoprotein involved in various physiological and pathological processes, including immune responses, inflammation, and tissue remodeling. In the CNS, SPP1 has been implicated in neuroinflammation and several neurological disorders. Recent studies indicate that SPP1 is significantly upregulated following IS, although its role appears context‐dependent. Some studies have also suggested a neuroprotective function, while others point to its detrimental effects [21, 22, 23]. Particularly relevant to our investigation, in AD models, SPP1 derived from perivascular cells induces microglial activation, driving aberrant synapse engulfment [16]. This raises an important question: Does SPP1 play a similar role in IS?

In this study, we hypothesized that SPP1 acts as a key regulator of microglia‐mediated synapse engulfment and GVU dysfunction after IS. To test this hypothesis, we employed a combination of in vivo middle cerebral artery occlusion/reperfusion (MCAO/R) mouse models with AAV‐mediated *Spp1* knockdown and in vitro oxygen–glucose deprivation/reperfusion (OGD/R) models with CRISPR/Cas9‐mediated *Spp1* knockout. Our results showed that SPP1 was significantly upregulated in the ischemic penumbra and that its reduction alleviated excessive synapse engulfment, preserved synaptic and GVU integrity, reduced neuroinflammation, and ultimately improved functional recovery. These findings position SPP1 as a critical driver of secondary injury following IS and a promising therapeutic target for preserving neural circuits.

## 2 Materials and Methods

### 2.1 Cell Culture

BV‐2 cells, Bend.3 cells, and HT‐22 cells were purchased from the cell bank of the Chinese Academy of Sciences. The BV‐2 cells were cultured in DMEM/F12 medium, while Bend.3 cells and HT‐22 cells were cultured in DMEM high‐glucose medium. The media were supplemented with 10% fetal bovine serum and 1% penicillin/streptomycin and cultured in an incubator at 37°C with 5% CO_2_.

### 2.2 Animals

Male C57BL/6 mice (2–3 months old, weighing 20–25 g) were used in this study. The mice were housed at the Neurology Institute of Anhui University of Chinese Medicine under standard laboratory conditions (12‐h light/dark cycle, temperature of 20°C–22°C, and humidity of 50%–60%), with access to food and water ad libitum. All experimental procedures were approved by the Animal Ethics Committee of Anhui University of Chinese Medicine.

### 2.3 Establishment of the MCAO/R Model

Focal cerebral ischemia was induced using the intraluminal filament method as previously described [24]. The mice were anesthetized using pentobarbital sodium (60 mg/kg). The right common carotid artery (CCA) was exposed via a midline cervical incision and ligated proximally, with the distal CCA temporarily secured near the bifurcation. Following external carotid artery (ECA) ligation and temporary clipping of the internal carotid artery (ICA), a silicone‐coated filament (CINOTECH, Beijing, China) was inserted through the CCA and advanced via the ICA to occlude the origin of the middle cerebral artery. After 1 h of ischemia, the filament was withdrawn to restore blood flow, and the incision was closed. Successful model establishment was confirmed 24 h postoperatively using neurological deficit scoring (1–3 on a 0–4 scale).

### 2.4 *Spp1* Knockdown Mouse Model Construction

An adeno‐associated virus (AAV) vector (HANBIO, Shanghai, China) carrying a short hairpin RNA (shRNA) targeting mouse *Spp1* (AAV‐*Spp1*‐shRNA) and a scrambled control shRNA (AAV‐scramble) was constructed. Thirty days prior to MCAO/R surgery, the study mice were anesthetized and placed in a stereotaxic frame. AAV vectors (3 μL) were injected into the ipsilateral temporoparietal cortex (coordinates relative to bregma: AP −2.0 mm, ML ±3.0 mm, DV −2.5 mm for cortex) at an infusion rate of 0.2 μL/min.

### 2.5 *Spp1* Knockout Glial‐Vascular Unit Model Construction

*Spp1* knockout was achieved via CRISPR/Cas9 gene editing. The gene knockout target is listed in Table S1. The resulting cells were exposed to oxygen–glucose deprivation/reperfusion (OGD/R) to induce an IS model and co‐cultured with BV‐2, Bend.3, and HT‐22 cells.

### 2.6 Super‐Resolution Vascular Imaging

After being anesthetized, each mouse was fixed in a prone position, and a cranial window was opened. Ultrasound gel was applied to the exposed area, and the target region was identified using the VINNO‐ULTIMUS 9LAB Small Animal Super‐Resolution Ultrasound System in 2D mode. A bolus of SonoVue (sulfur hexafluoride microbubbles) was injected via the tail vein, and imaging was initiated immediately to acquire parameters such as Den, Dir, and Vel.

### 2.7 Laser Speckle Contrast Imaging

Following MCAO/R, cerebral blood perfusion was assessed using a laser speckle blood monitor. The mice were anesthetized using 2% isoflurane, and a charge‐coupled device camera was positioned ~11 cm above the head. A laser diode illuminated the intact skull, and laser speckle imaging was performed with a resolution of 1544 × 2064 pixels for 5 min. The average blood perfusion value was also calculated.

### 2.8 Behavioral Assessment

#### 2.8.1 Open‐Field Test

Locomotor activity was evaluated in a 50 × 50 × 40‐cm arena that was divided into central and peripheral zones. Time spent, crossings, and rearing events were recorded during acclimation trials (the box was cleaned with 75% ethanol between sessions).

#### 2.8.2 Barnes Maze

Spatial memory was tested using a circular platform (diameter of 122 cm and height of 99 cm) with 20 escape holes. Mice underwent 4 days of training followed by probe testing on day 5, with escape latency recorded via video tracking.

#### 2.8.3 Rotarod Test

Motor coordination was assessed over three consecutive days (three trials/day, 5 min/trial) with acceleration from 5 to 40 rpm. Latency to fall was recorded and averaged across the trials.

### 2.9 Transmission Electron Microscopy (TEM)

Brain tissues were sliced into 1‐mm^3^ sections and fixed in 2.5% glutaraldehyde (Service bio, Wuhan, China) overnight at 4°C. After post‐fixation in 1% osmic acid (Ted Pella Inc., California, USA) for 2 h, the tissue was dehydrated, embedded in resin, and sectioned into 60–80‐nm‐thick slices for examination under a HITACHI HT7800/HT7700 transmission electron microscope.

### 2.10 Fluorescent Staining

Fluorescent staining was performed after transcardial perfusion and overnight fixation in 4% paraformaldehyde (Servicebio, Wuhan, China). Brain sections (20–30 μm thick) were incubated with target antibody overnight at 4°C, followed by secondary antibody incubation and visualization using a NIKON ECLIPSE C1 microscope.

### 2.11 TUNEL Staining

Apoptotic neurons were identified using the Fluorescein TUNEL Cell Apoptosis Detection Kit (Servicebio, Wuhan, China) according to the manufacturer's instructions. The samples were visualized using an Olympus BX53 microscope.

### 2.12 Quantitative Real‐Time PCR (RT‐qPCR)

Total RNA was extracted from cells and ischemic penumbra tissues using the TRIZOL method. cDNA was synthesized using the SPARKscript II RT Plus Kit (AG0304‐B, SparkJade), and PCR was performed to measure mRNA levels (Table S2). qPCR conditions included an initial denaturation at 94°C for 3 min, followed by 40 cycles at 94°C for 10 s and 60°C for 34 s. The results were normalized to β‐actin and presented as the 2^−ΔΔCt^ value.

### 2.13 Western Blotting

Proteins were extracted from brain tissues and cells using RIPA buffer (R0020, Solarbio) and quantified using the BCA assay (P0010, Beyotime). SDS‐PAGE electrophoresis was performed, and proteins were transferred to nitrocellulose membranes. The membranes were blocked, incubated with primary antibodies (Table S3), and then with horseradish peroxidase‐conjugated secondary antibodies. Protein bands were detected using a gel imaging system.

### 2.14 Statistical Analyses

Data are presented as mean ± SEM. Statistical analyses were performed using GraphPad Prism 9 (GraphPad, La Jolla, CA, USA). Normality was confirmed using the Shapiro–Wilk test (*p* > 0.05), and homogeneity of variance was verified using the Brown–Forsythe test (*p* > 0.05); therefore, parametric statistical methods were employed. Comparisons between two groups were made using an independent sample *t*‐test. Comparisons among multiple groups were performed using one‐way ANOVA with Tukey's post hoc test. A *p* < 0.05 was considered statistically significant.

## 3 Results

### 3.1 Microglia‐Mediated Synapse Engulfment and Synaptic Damage in the Ischemic Penumbra

**FIGURE 1:**
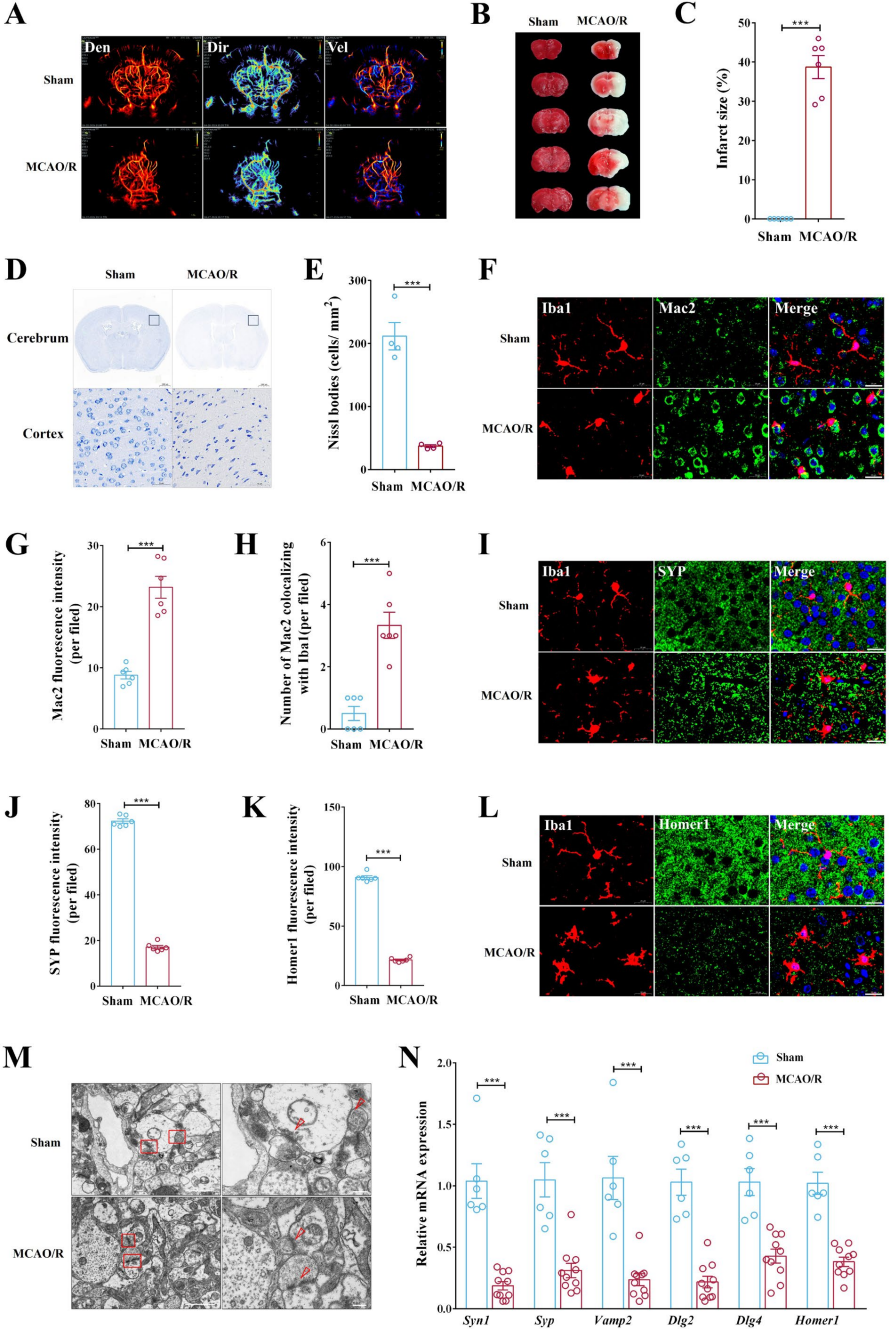
Excessive microglia‐mediated synapse engulfment and synaptic damage occur in the ischemic penumbra of the MCAO/R mouse model. (A) The density, direction, and velocity of cerebral blood flow were measured using a small animal super‐resolution ultrasound imaging system (*n* = 3 per group). (B) Representative TTC images of infarction. (C) Infarct volume (*n* = 6 per group; ***p < 0.001). (D) Representative Nissl images. The lower images are enlarged versions of the black boxes on the upper images. Scale bars on the upper represent 1000 μm, and those on the lower represent 50 μm. (E) The number of Nissl bodies (*n* = 4 per group; ***p < 0.001). (F) Representative immunofluorescence double‐staining images of Iba1/Mac2. Scale bar, 20 μm. (G, H) Mac2 fluorescence intensity (per field) (G). The number of Mac2 colocalizing with Iba1 (H) (*n* = 6 per group; ***p < 0.001). (I, J) Representative immunofluorescence double‐staining images of Iba1/SYP. Scale bar, 20 μm (I). The fluorescence intensity of Syp (*n* = 6 per group; ***p < 0.001) (J). (K, L) The fluorescence intensity of Syp (*n* = 6 per group; ***p < 0.001) (K). Representative immunofluorescence double‐staining images of Iba1/Homer1. Scale bar, 20 μm (L). (M) Representative images of TEM. The right‐side images are enlarged versions of the red boxes in the left images, and the arrow points to synaptic vesicles and the pre‐ and postsynaptic membranes. Scale bars on the left represent 2.0 μm, and those on the right represent 500 nm. (N) RT‐qPCR was used to measure the mRNA expression levels of synapse‐related genes in the ischemic penumbra (*n* = 6–10 per group; ***p < 0.001). Independent samples *T*‐test; data are presented as mean ± SEM.

To investigate the pathological alterations following IS, a transient middle cerebral artery occlusion/reperfusion (MCAO/R) mouse model was established. Successful occlusion and reperfusion were confirmed by a marked reduction in cerebral blood flow in the MCAO/R group, as compared to the sham‐operated group, measured using a small animal super‐resolution ultrasound imaging system (Figure 1A). Triphenyl tetrazolium chloride (TTC) staining, performed 24 h post‐reperfusion, revealed a significant infarct volume in the MCAO/R mice, which was absent in the sham group (Figure 1B,C). Similarly, Nissl staining showed a pronounced reduction in the number of surviving neurons within the ischemic penumbra of the temporo‐parieto‐occipital cortex, indicating substantial neuronal injury (Figure 1D,E).

Considering the role of microglia in post‐ischemic brain injury, we evaluated their phagocytic activity. Immunofluorescence staining for Iba1 (a microglial marker) and Mac2 (a marker of phagocytic microglia/macrophages) demonstrated a substantial increase in Mac2 expression and co‐localization with Iba1 in the ischemic penumbra of MCAO/R mice, indicating an elevated phagocytic response (Figure 1F–H). We also observed marked loss of presynaptic (SYP) and postsynaptic (Homer1) markers, with reduced co‐localization with microglia consistent with active synaptic engulfment (Figure 1I–L). Ultrastructural analysis using transmission electron microscopy (TEM) confirmed synaptic damage, including reduced vesicle density, narrowed synaptic clefts, and indistinct membrane boundaries (Figure 1M). RT‐qPCR analysis further revealed significant downregulation of synapse‐related genes (*Syn1, Syp, Vamp2, Dlg2, Dlg4/Psd95, Homer1*, etc.) (Figures 1N and S1). These findings indicate that excessive microglial phagocytosis drives synaptic degradation following MCAO/R, prompting us to identify the key molecular mediators of this pathological process.

### 3.2 Proteomic Analysis Identifies SPP1, a Perivascular Signaling, as a Key Upregulated Protein in the Ischemic Penumbra

**FIGURE 2:**
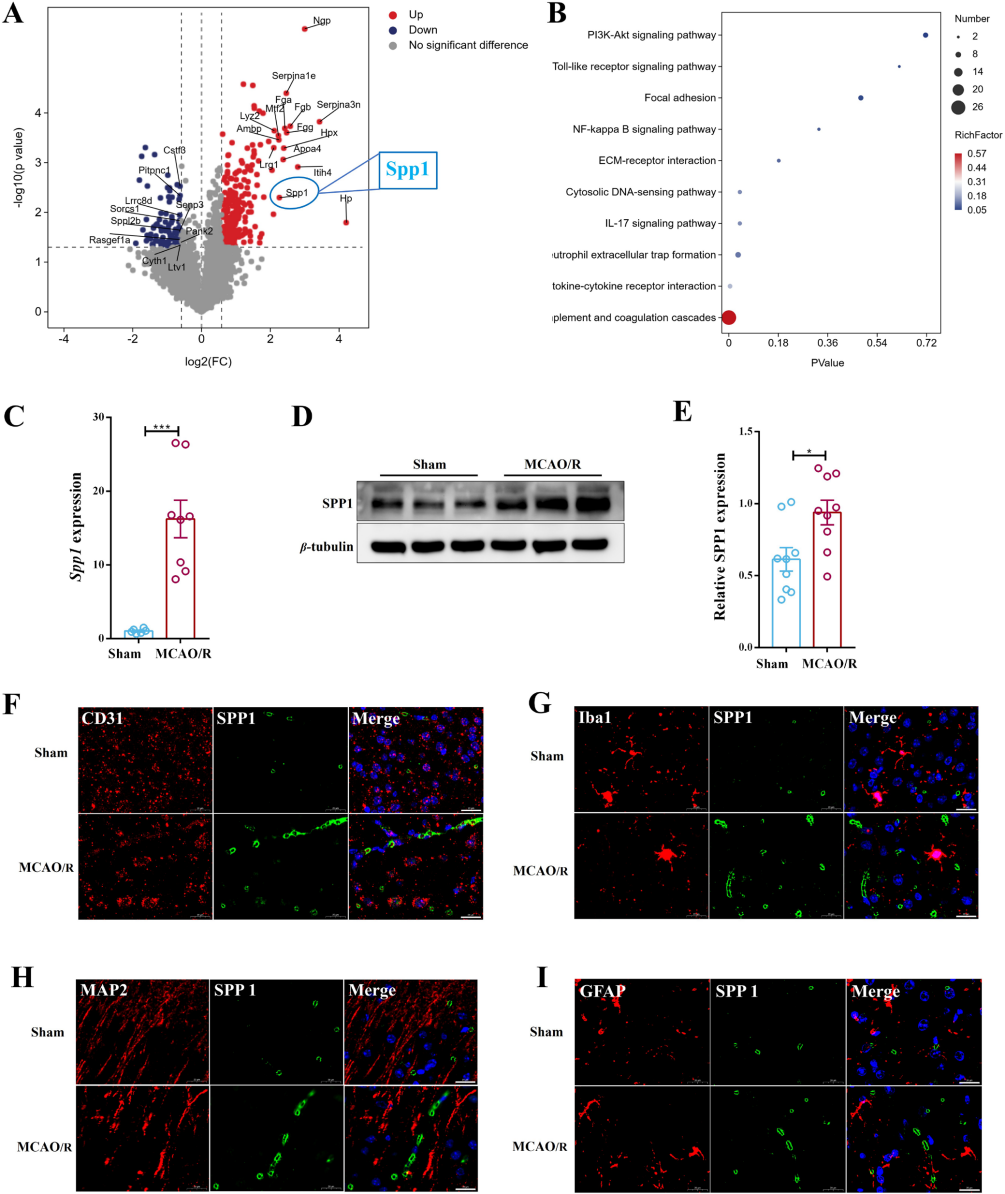
Proteomics identified SPP1 as a differentially expressed protein in the ischemic penumbra of the MCAO/R mouse model, enriched in the perivascular space. (A, B) Volcano plot (A); KEGG pathway enrichment analysis (B) (*n* = 4 per group). (C–E) RT‐qPCR and Western blot further confirmed that *Spp1* (C) (*n* = 8 per group; ***p < 0.001) and protein expression were markedly elevated in the ischemic penumbra of the MCAO/R group compared to the Sham group (D, E) (*n* = 9 per group; *p < 0.05). (F–I) Representative immunofluorescence double‐staining images for CD31/SPP1, Iba1/SPP1, MAP2/SPP1, and GFAP/SPP1. Scale bar, 20 μm. Independent samples *T*‐test; data are presented as mean ± SEM.

To identify molecular drivers of the microglial‐mediated synaptic damage observed, we conducted label‐free quantitative proteomics on tissue samples from the ischemic penumbra of both sham and MCAO/R mice. The analysis revealed numerous differentially expressed proteins (Figure S2A,B), with SPP1 being one of the most significantly upregulated proteins (Figure 2A). The KEGG and Gene Ontology enrichment analyses indicated significant enrichment in pathways related to inflammation, immune response, and vascular function, while SPP1 was implicated in several of these pathways (Figures 2B and S2C).

Validation by RT‐qPCR and Western blot confirmed robust upregulation of SPP1 mRNA and protein in the penumbra (Figure 2C–E). Cellular localization studies also revealed that SPP1 co‐localizes extensively with the endothelial cell marker CD31, indicating its presence in or around blood vessels (Figure 2F). Additionally, SPP1 co‐localized with the microglial marker Iba1 (Figure 2G), while showing minimal overlap with the neuronal marker MAP2 and the astrocyte marker GFAP (Figure 2H,I). These results suggest that SPP1 is significantly upregulated in the ischemic penumbra after stroke and is primarily associated with microglia and the vasculature, implicating it as a potential mediator of microglia–vascular interactions and microglial phagocytosis.

### 3.3 *Spp1* Knockout Modulates Neuroinflammation and Exerts Neuroprotective Effects in an In Vitro Ischemia Model

Based on SPP1 upregulation in microglia and vasculature, we hypothesized that SPP1 promotes the observed pathological microglial activation and synaptic loss. To test this hypothesis, we generated *Spp1* knockout (*Spp1* ^−/−^) cells using CRISPR/Cas9 gene editing and subjected them to an in vitro ischemia model, oxygen–glucose deprivation/reperfusion (OGD/R). Successful knockout was confirmed at both the mRNA and protein levels (Figure S3A–E).

**FIGURE 3:**
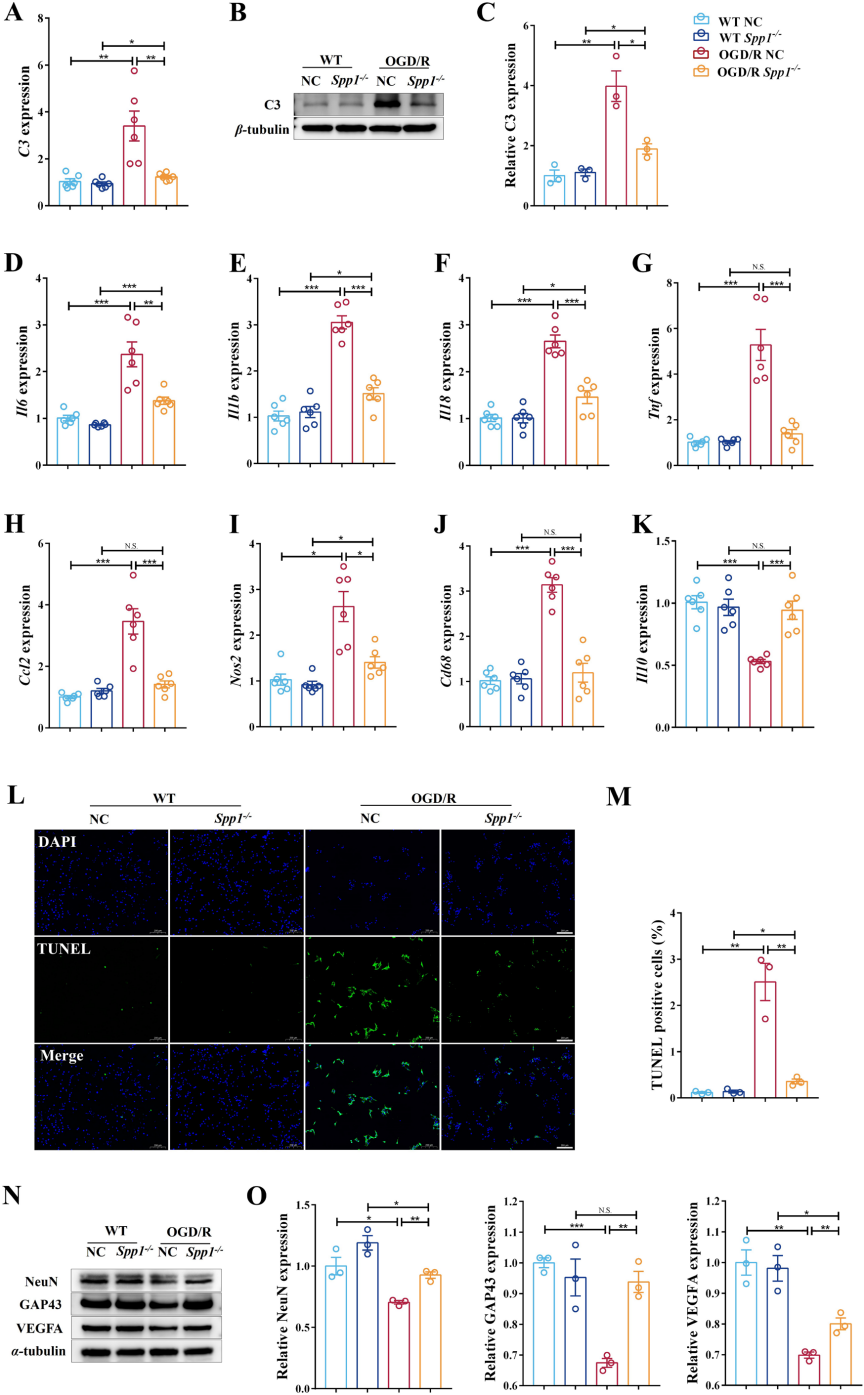
Bend.3 *Spp1* ^*−/−*^ modulates neuroinflammation, maintains homeostasis during the immune response, and exerts neuroprotective effects in the OGD/R model. (A) RT‐qPCR was used to quantify the expression level of *C3* in BV‐2 co‐cultured with Bend.3 and HT‐22 (*n* = 6 per group; *p < 0.05, **p < 0.01). (B, C) Gray‐scale band image of C3 (B). Expression level of C3 was quantified using Western blot (*n* = 3 per group; *p < 0.05, **p < 0.01) (C). (D–K) Expression levels of *Il10, Il6, Il1b, Il18, Tnf, Ccl2, Nos2, and Cd68* in BV‐2 co‐cultured with Bend.3 and HT‐22 were quantified using RT‐qPCR (*n* = 6 per group; *p < 0.05, **p < 0.01, ***p < 0.001) (L) Images of TUNEL immunofluorescence staining in HT‐22 co‐cultured with Bend.3 and BV‐2. Scale bar, 20 μm. (M) The TUNEL‐positive cells per field (*n* = 3 per group; ***p < 0.001). (N) Gray‐scale band image of NeuN, GAP43, and VEGF in HT‐22 co‐cultured with Bend.3 and BV‐2. (O) The protein quantification of NeuN, GAP43, and VEGF were performed using Western blot (*n* = 3 per group; *p < 0.05, **p < 0.01, ***p < 0.001). One‐way ANOVA with Tukey's post hoc test; Data are presented as mean ± SEM.

We evaluated the impact of *Spp1* knockout on neuroinflammation. The complement system, a key mediator of inflammation and synapse elimination, was examined. OGD/R induced a significant upregulation of complement component C3, which was substantially attenuated in *Spp1* ^−/−^ cells (Figure 3A–C). Afterwards, we assessed the expression of various pro‐inflammatory and anti‐inflammatory cytokines. *Spp1* knockout significantly reduced the OGD/R‐induced increase in pro‐inflammatory mediators, including *Il1b, Il18, Tnf, Ccl2*, and the microglial activation marker *Cd68* (Figure 3D–J). In contrast, the expression of the anti‐inflammatory cytokine *Il10* was significantly higher in *Spp1* ^−/−^ cells compared to wild‐type cells following OGD/R (Figure 3K). These findings were further corroborated by ELISA measurements of cytokine proteins in the culture medium (Figure S4A–D). Furthermore, we examined whether the anti‐inflammatory effects of *Spp1* knockout translated into neuroprotection. Functionally, *Spp1* deficiency markedly reduced apoptotic cell death (TUNEL staining) (Figure 3L,M) and preserved neuronal markers (NeuN, GAP43) while upregulating VEGF (Figure 3N,O). These in vitro findings strongly suggest that SPP1 promotes a pro‐inflammatory environment and exacerbates neuronal death after ischemic injury, while its absence facilitates a more balanced immune response and enhances neuronal survival, indicating that it is a suitable therapeutic target.

### 3.4 AAV‐Mediated *Spp1* Knockdown Improves Functional Recovery and Reduces Neuronal Damage in the MCAO/R Mouse Model

**FIGURE 4:**
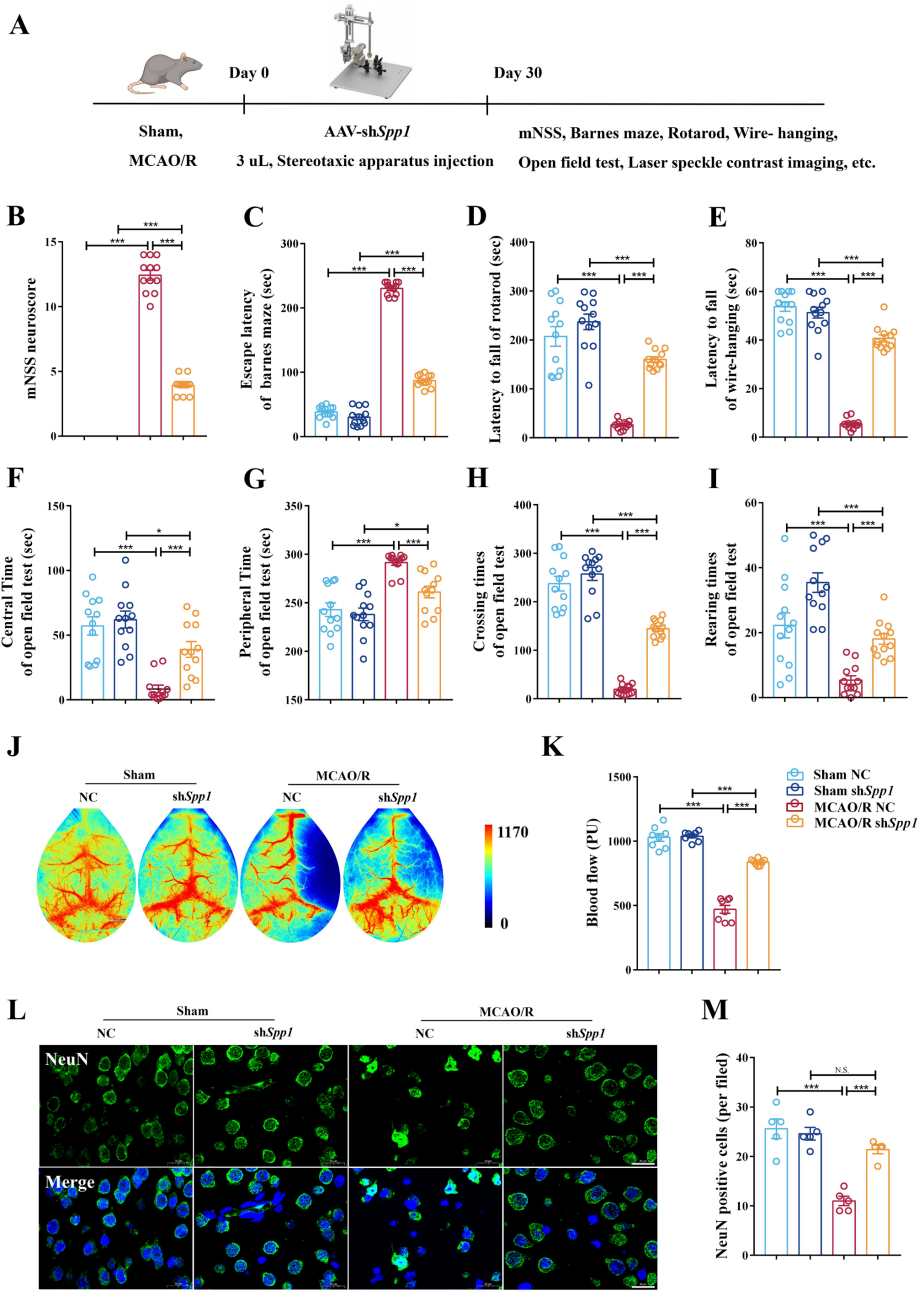
sh*Spp1* provides protection against behavior impairment and alleviates neuronal damage in the ischemic penumbra of the MCAO/R mouse model. (A) Overall experimental flowchart. (B) The mNSS score after sh*Spp1* treatment (*n* = 12 per group; ***p < 0.001). (C) Barnes maze after sh*Spp1* treatment (*n* = 12 per group; ***p < 0.001). (D) The rotarod test after sh*Spp1* treatment (*n* = 12 per group; ***p < 0.001). (E) Wire‐hanging test sh*Spp1* treatment (*n* = 12 per group; ***p < 0.001). (F–I) Time traveled in the center zone (F), time traveled in the peripheral zone (G), crossing times (H), and rearing times (I) were observed using an open field test (*n* = 12 per group; *p < 0.05, ***p < 0.001). (J) Laser speckle contrast imaging. (K) Blood perfusion in brain hemispheres after sh*Spp1* in the different groups (*n* = 8 per group; One‐way ANOVA with Tukey's post hoc test; ***p < 0.001). (L, M) Images of NeuN in the brain hemispheres. Scale bar, 20 μm (L). NeuN cells per field in the brain hemispheres (*n* = 5 per group; ***p < 0.001) (M). One‐way ANOVA with Tukey's post hoc test; Data are presented as mean ± SEM.

To translate our in vitro findings to an in vivo context, we stereotaxically injected AAV expressing *Spp1*‐targeting shRNA (sh*Spp1*) or a non‐targeting control (NC) prior to MCAO/R (Figure 4A). The efficacy of the AAV‐mediated *Spp1* knockdown was confirmed in the ischemic penumbra (Figure S5A–E).

MCAO/R‐induced significant neurological deficits, as assessed by the modified neurological severity score (mNSS), were markedly improved in sh*Spp1*‐treated mice (Figure 4B). In the Barnes maze test for spatial learning and memory, MCAO/R NC mice exhibited prolonged escape latencies compared to sham mice, whereas sh*Spp1* mice demonstrated significantly enhanced performance (Figure 4C). Motor coordination and strength were evaluated using the rotarod and wire‐hanging tests. *Spp1* knockdown notably improved latency to fall in both tests compared to the MCAO/R NC group (Figure 4D,E). The open field test revealed MCAO/R‐induced anxiety‐like behaviors, characterized by reduced time spent in the center zone and fewer crossings and rearing events, all of which were partially reversed by sh*Spp1* treatment (Figure 4F–I).

To investigate the physiological basis for these functional improvements, we assessed cerebral blood perfusion using laser speckle contrast imaging. MCAO/R led to a sustained reduction in blood flow in the affected hemisphere, which was significantly restored in the sh*Spp1*‐treated group, indicating improved microvascular function (Figure 4J,K). Consistent with these findings and our in vitro data, immunofluorescence staining for NeuN showed that *Spp1* knockdown significantly protected against neuronal loss in the ischemic penumbra (Figure 4L,M). Given the observed vascular perfusion improvements, we next investigated whether SPP1 regulates blood–brain barrier integrity.

### 3.5 SPP1 Modulates the Integrity of the BBB and Glial‐Vascular Unit (GVU) Homeostasis

**FIGURE 5:**
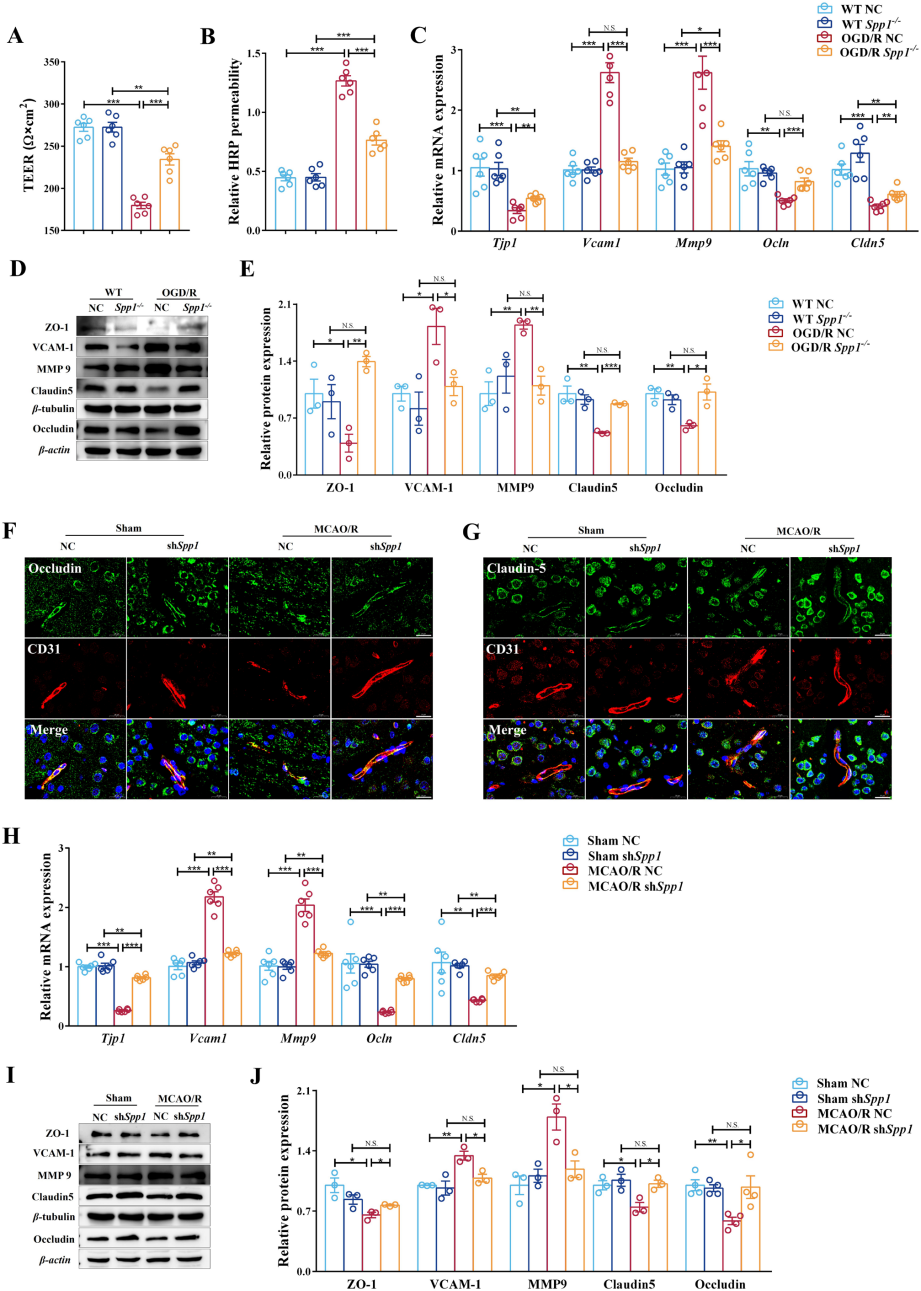
*Spp1* modulates BBB homeostasis. (A, B) Effects of *Spp1* on barrier function in HT‐22 co‐cultured with Bend.3 and BV‐2 of OGD/R model, including Time‐course of TEER (A), and HRP flux (B) (*n* = 6 per group; **p < 0.01, ***p < 0.001). (C–E) RT‐qPCR was used to quantify mRNA levels of *Tjp1*, *Vcam1*, *Mmp9*, *Ocln*, and *Cldn5* in HT‐22 co‐cultured with Bend.3 and BV‐2 of OGD/R model (C) (*n* = 6 per group; *p < 0.05, **p < 0.01, ***p < 0.001). Gray‐scale band images (D). Expression levels of ZO‐1, VCAM‐1, MMP9, claudin5, and occludin were quantified using Western blot in the different groups (E) (*n* = 3 per group; *p < 0.05, **p < 0.01, ***p < 0.001). (F, G) Representative fluorescence images of the TJ protein occludin or claudin‐5 and CD31. Scale bar, 20 μm. (H–J) RT‐qPCR was used to quantify mRNA levels of *Tjp1*, *Vcam1*, *Mmp9*, *Ocln*, and *Cldn5* in the ischemic penumbra of the different groups (H) (*n* = 6 per group; **p < 0.01, ***p < 0.001). Gray‐scale band images (I). Levels of ZO‐1, VCAM‐1, MMP9, claudin‐5, and occludin were quantified using Western blot (*n* = 3 per group; *p < 0.05, **p < 0.01, ***p < 0.001) (J). One‐way ANOVA with Tukey's post hoc test; data are presented as mean ± SEM.

The observed improvement in cerebral perfusion following *Spp1* knockdown suggests that SPP1 plays a role in regulating the integrity of the GVU and the BBB. To further investigate this, we assessed the expression of the permeability of the BBB and key BBB components both in vivo and in vitro. In vitro, OGD/R injury markedly decreased TEER and increased HRP compared with the normal group. After *Spp1* knockout treatment, TEER recovered to 86.04% of the normal value and HRP leakage dropped to 60.26% of the injured level, indicating that *Spp1* knockout restores OGD/R‐induced barrier dysfunction (Figure 5A,B). Surprisingly, we found that similar changes in the expression of GVU‐related genes and proteins were induced, and these were significantly attenuated in *Spp1* ^−/−^ cells (Figure 5C–E). In vivo, MCAO/R disrupted vascular morphology and tight junction integrity (segmental loss of occludin and claudin‐5, tortuous CD31‐positive vessels), whereas sh*Spp1* treatment restored linear vascular architecture and continuous tight junction staining (Figure 5F,G). RT‐qPCR and Western blot analysis confirmed that *Spp1* knockdown preserved tight junction genes (*Tjp1, Ocln, and Cldn5*) and proteins (ZO‐1, claudin‐5, and occludin) while it reduced the expression levels of BBB disruption markers (VCAM1 and MMP9) at both the mRNA and protein levels (Figure 5H–J). These consistent findings across both in vivo and in vitro models indicate that SPP1 contributes to the disruption of GVU homeostasis and BBB integrity following ischemic injury. Reducing SPP1 levels appears to better preserve the stability of the vascular unit, which likely facilitates the microglial synaptic engulfment observed as above.

### 3.6 *Spp1* Knockdown Attenuates Microglia‐Mediated Synapse Engulfment and Preserves Synaptic Integrity

**FIGURE 6:**
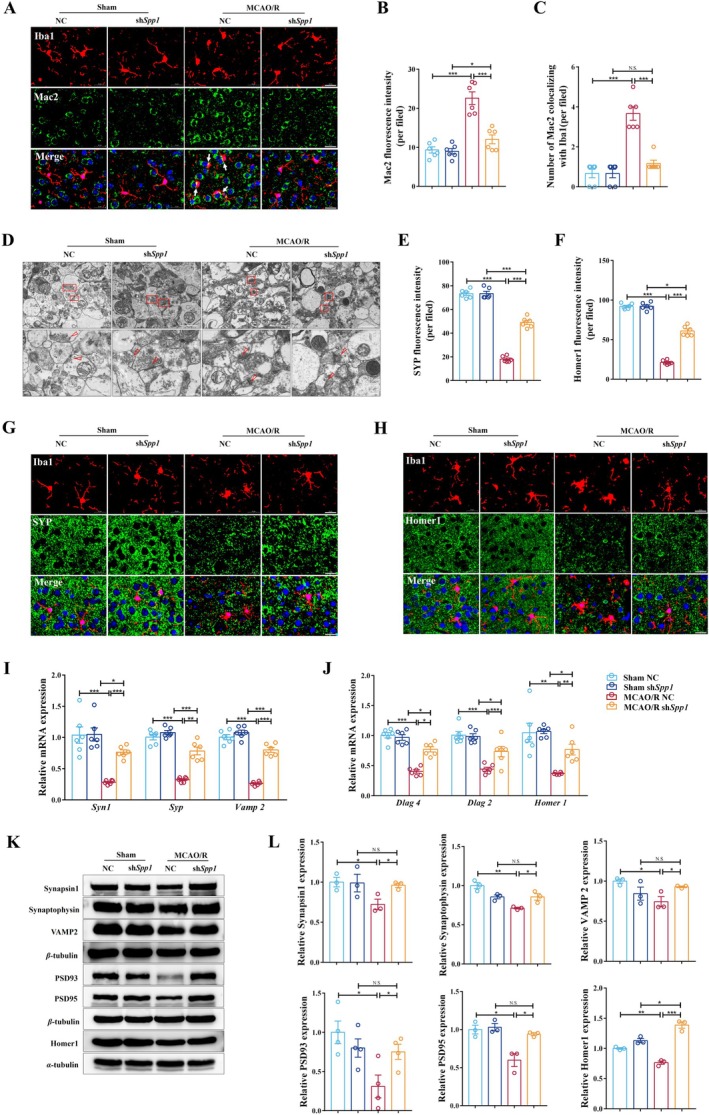
*Spp1* suppresses synapse engulfment and consequently improves synaptic function in the ischemic penumbra of the MCAO/R mouse model. (A–C) Representative immunofluorescence images for Iba1 colocalizing with Mac2 in the ischemic penumbra after sh*Spp1*in the different groups. Scale bar, 20 μm (A). Mac2 fluorescence intensity (B). The number of Mac2 colocalizing with Iba1 (C) (*n* = 6 per group; *p < 0.05, ***p < 0.001). (D) Representative images of TEM. The lower images are enlarged versions of the red boxes on the upper images, and the arrow points to synaptic vesicles and the pre‐ and postsynaptic membranes. Scale bars on the left represent 2.0 μm, and those on the right represent 500 nm. (E–H) The fluorescence intensity of SYP (*n* = 6 per group; ***p < 0.001) (E). The fluorescence intensity of Homer 1 (*n* = 6 per group; *p < 0.05, ***p < 0.001) (F). Representative immunofluorescence double‐staining images for Iba1 colocalizing with SYP. Scale bar, 20 μm (G). Representative immunofluorescence double‐staining images for Iba1 colocalizing with Homer1. Scale bar, 20 μm (H). (I, J) RT‐qPCR was used to measure the mRNA expression levels of *Syn1*, *Vamp2*, *Syp*, *Dlag2*, *Dlag4*, and *Homer1* in the ischemic penumbra of the different groups. (*n* = 6 per group; *p < 0.05, **p < 0.01, ***p < 0.001). (K, L) Gray‐scale band image (K). Expression levels of Synapsin1, VAMP2, Synaptophysin, PSD93, PSD95, and Homer1 quantified using Western blot (*n* = 3 per group; *p < 0.05, **p < 0.01, ***p < 0.001) (L). One‐way ANOVA with Tukey's post hoc test; data are presented as mean ± SEM.

Having established that SPP1 compromises vascular integrity and drives inflammation, we redirected our attention to the initial observations of the central pathological features of excessive microglial phagocytosis and synaptic loss. In vivo, sh*Spp1* significantly reduced Mac2 upregulation in Iba1‐positive microglia (Figure 6A–C). TEM analysis revealed that *Spp1* knockdown preserved ultrastructural synaptic integrity, thereby maintaining vesicle density and membrane definition at levels comparable to those of sham controls (Figure 6D). Immunofluorescence confirmed that *Spp1* knockdown preserved SYP and Homer1 density while reducing their co‐localization with microglia (Figure 6E–H). At the molecular level, sh*Spp1* prevented downregulation of presynaptic (*Syn1, Syp*, and *Vamp2*) and postsynaptic (*Dlg2, Dlg4*, and *Homer1*) genes (Figures 6I,J and S6) and proteins (Synapsin1, PSD95, etc.) (Figure 6K,L). To confirm that these effects were directly attributable to the absence of SPP1 in ischemia, we utilized our in vitro OGD/R model. Consistent with the in vivo findings, *Spp1* knockout prevented the OGD/R‐induced downregulation of the same synaptic genes and proteins (Figures S7A–C and S8A–J). Collectively, these findings complete the mechanistic framework, demonstrating that SPP1 inhibition disrupts the pathological axis of vascular dysfunction, microglial hyperactivation, and synaptic engulfment, thus preserving synaptic integrity and breaking the cycle of neurovascular dysfunction in IS.

## 4 Discussion

In this study, we elucidated the critical role of perivascular‐derived signaling SPP1 in the pathophysiology of IS, specifically in promoting excessive microglia‐mediated synapse engulfment and disrupting the homeostasis of the GVU, acting as the trigger linking BBB leakage to microglia‐mediated synapse engulfment. We found that ischemic injury induces a dramatic upregulation of SPP1 in the ischemic penumbra, which drives a pro‐inflammatory and phagocytic microglial phenotype. This consequently leads to the pathological removal of synapses, breakdown of the BBB, and exacerbation of neuronal injury. Importantly, genetic knockdown of *Spp1* in vivo or knockout in vitro reversed these detrimental effects, preserving synaptic integrity, stabilizing GVU function, and significantly improving neurological outcomes.

The dual nature of microglial activation in response to brain injury is well established. While microglial phagocytosis is essential for clearing dead cells and debris, its excessive activation can be highly detrimental [25, 26]. Our results align with growing evidence indicating that, in acute brain injuries, including stroke, microglia may excessively target and eliminate synapses that are stressed but could otherwise recover [13, 14, 27]. A key challenge in the field has been identifying the molecular switches that drive microglial responses from protective to pathological. Our study identifies SPP1 as one such critical switch.

The role of SPP1 in CNS pathologies is complex and context‐dependent. While some studies have suggested a neuroprotective role for SPP1, particularly through inhibiting pyroptosis [28], our findings are consistent with those of reports linking elevated SPP1 to negative outcomes in stroke and neurodegenerative diseases [20, 21]. Specifically, SPP1 has been implicated in exacerbating ischemic injury by promoting ferroptosis and has been identified as a key signal in AD models, driving microglia to engulf synapses [16]. Our data strongly support this latter mechanism in IS, where we found that SPP1 is primarily localized to microglia and vasculature. Knockdown of SPP1 significantly reduced the phagocytic marker Mac2 and preserved synaptic structures, indicating that SPP1, whether acting autocrine or paracrine, is a key signal that activates microglia to engage in excessive synapse removal.

The mechanism by which SPP1 promotes synapse engulfment likely involves multiple pathways. During ischemic injury, upregulated SPP1 engages these receptors on microglia, initiating interconnected signaling pathways that drive the pro‐phagocytic phenotype.

Through CD44, SPP1 activates the PI3K/AKT axis, triggering MAPK cascades (p38 MAPK and ERK1/2) that converge on NF‐κB pathway activation [29, 30]. Activated NF‐κB transcriptionally upregulates pro‐inflammatory cytokines, phagocytic receptors, and complement components. A critical downstream consequence of SPP1 signaling is the modulation of the complement cascade, which serves as an executioner mechanism for synapse elimination. In AD models, SPP1 has been shown to upregulate microglial expression of complement component C1qa, which is the initiating molecule of the classical complement pathway [16]. Our finding that Spp1 knockout reduced C3 expression in response to oxygen–glucose deprivation/reperfusion (OGD/R) provides direct evidence linking SPP1 to complement activation in ischemia. We propose that SPP1 acts upstream to amplify the complement cascade. According to established literature, SPP1‐CD44/integrin signaling would enhance C1qa expression, thereby initiating the classical complement pathway: C1q‐mediated opsonization followed by sequential cleavage of C4 and C2 to form the C3 convertase. This enzymatic complex cleaves C3 into C3a and C3b, with C3b deposition serving as the critical “eat‐me” signal for microglial complement receptor 3 (CR3) [31]. In this working model, CR3 engagement by C3b‐opsonized synapses triggers the intracellular signaling required for actin polymerization and phagocytic engulfment. Therefore, our data support a model in which SPP1 establishes a molecular bridge from initial ischemic insult to complement‐mediated synapse elimination through coordinated receptor signaling.

A novel aspect of our study is the connection between SPP1, synapse engulfment, and the stability of the GVU. We demonstrated that Spp1 knockdown not only preserved synapses but also maintained the expression of key BBB tight junction proteins while reducing markers of vascular inflammation and degradation, such as VCAM‐1 and MMP9. These findings are consistent with those of studies that identified SPP1 as a highly upregulated factor in all neurovascular unit cell types after stroke, contributing to BBB breakdown. The link between synaptic health and vascular integrity is bidirectional: BBB disruption allows inflammatory molecules and peripheral immune cells to enter the brain, which further activates microglia and promotes synapse loss. Conversely, loss of synaptic and neuronal support can compromise the function of adjacent vascular cells. By targeting SPP1, we interrupt this vicious cycle, protecting both the synaptic and vascular components of the GVU.

From a therapeutic perspective, our findings are highly significant. Current acute stroke treatments are limited to reperfusion therapies, such as thrombolysis and mechanical thrombectomy, which must be administered within a narrow time window and fail to address secondary injury cascades. Our findings suggest that inhibiting SPP1 could serve as a potent neuroprotective strategy, complementing reperfusion therapies. By restraining excessive microglial phagocytosis, an SPP1‐targeted therapy could help preserve neural circuits in the ischemic penumbra, potentially leading to improved long‐term functional recovery for a broader range of patients with stroke.

Although our study provides valuable insights, it has several limitations that must be addressed. We demonstrated the neuroprotective effects of SPP1 knockdown in male mice; however, the degree of protection likely varies according to sex and age. Young female subjects exhibit estrogen‐mediated baseline neuroprotection that may attenuate the incremental benefit of SPP1 inhibition, whereas aged female subjects, that have lost estrogen protection and display X‐chromosome‐mediated pro‐inflammatory priming, may represent the population with the greatest therapeutic potential for SPP1‐targeted therapy [32, 33, 34, 35, 36, 37]. Therefore, direct comparative studies across sexes and age groups are essential. However, several caveats must be addressed before translating these stratified approaches to human stroke pathology. First, our experiments were conducted in mouse models, and the applicability of these sex‐specific findings to human conditions requires validation. Furthermore, while we identified SPP1 as a key driver of pathological processes, the precise downstream signaling pathways within microglia that mediate its effects on phagocytosis and inflammation post‐stroke require further exploration. Future studies should also investigate the relative contributions of SPP1 from different cellular sources, such as microglia versus perivascular cells, to fully understand its role in stroke pathology.

## 5 Conclusion

Our study identified perivascular‐derived signaling SPP1 as a critical mediator of secondary brain injury following IS and as a trigger linking BBB leakage to microglia‐mediated synapse engulfment. We demonstrated that the perivascular‐derived signaling was significantly upregulated in the ischemic penumbra, where it promoted a detrimental, hyperphagocytic microglial state. This upregulation could lead to excessive synapse engulfment, disruption of the GVU, and exacerbation of neuronal damage, ultimately impairing functional recovery. By inhibiting the perivascular‐derived signaling, we were able to restrain this pathological microglial activity, preserve synaptic and vascular integrity, and significantly improve neurological outcomes in mouse stroke models. These findings reveal a key mechanism of post‐stroke neurodegeneration and position the perivascular‐derived signaling SPP1 as a promising therapeutic target for the development of novel neuroprotective strategies for patients with IS.

## Author Contributions

Chenchen Xu: conceptualization, animal experiments, cellular experiments, data curation, writing – original draft. Xiaoxiao Li, Nan Cheng, Rui Zhao, Xin Wang, and Wenxin Xia: animal experiments. Jianjian Dong and Yongsheng Han: supervision, writing – review and editing. All authors discussed the results and reviewed the manuscript.

## Funding

This work was supported by National Natural Science Foundation of China (grant numbers: 82274656 and 82575236), Exploratory Science Foundation of Anhui University of Chinese Medicine (grant number: AHUCM2024TS128), and Program for Excellent Sci‐tech Innovation Teams of Anhui Provincial Department of Education (grant number: 2025AHGXZK10040).

## Ethics Statement

All experimental procedures were approved by the Animal Ethics Committee of Anhui University of Chinese Medicine (AHUCM‐mouse‐2023131).

## Conflicts of Interest

The authors declare no conflicts of interest.

## Supporting information


**Table S1:** Primers used for *Spp1* knockout via CRISPR/Cas9 gene editing.
**Table S2:** Primers used for qPCR.
**Table S3:** Antibody used for western blot.
**Figure S1:** Evidence of synaptic deficits in the ischemic penumbra of the MCAO/R mouse model. (A–C) RT‐qPCR was used to quantify mRNA levels of presynaptic, postsynaptic, and active‐zone‐related genes in the ischemic penumbra of the Sham and MCAO/R groups (*n* = 6–10 per group; **p < 0.01, ***p < 0.001). Independent samples *T*‐test; data are presented as mean ± SEM.
**Figure S2:** (A) Venn diagrams. (B) Heatmap of differentially expressed proteins in the Sham and MCAO/R groups. (C) Gene Ontology Enrichment of Spp1.
**Figure S3:** Verification of Spp1−/− in Bend.3 of OGD/R model. (A) CRISPR/Cas9 gene editing technology was used to induce Spp1−/− in vitro. The expression level of Spp1 was quantified using RT‐qPCR in different groups (*n* = 6 per group; ***p < 0.001). (B, C) Western blot was used to quantify SPP1 protein level (*n* = 3 per group; *p < 0.05). (D) Representative immunofluorescence image of SPP1. Scale bar, 20 μm. (E) The ratio of SPP1 fluorescence intensity to the nucleus (*n* = 3 per group; ***p < 0.001). One‐way ANOVA with Tukey's post hoc test; data are presented as mean ± SEM.
**Figure S4:** Bend.3 Spp1−/− modulate neuroinflammation in OGD/R model. (A–D) Microplate reader results showing IL‐1β (A), TNF‐α (B), IL‐10 (C), and TNF‐β (D) activities in the culture medium of Bend.3 co‐cultured with BV‐2 and HT‐22 (*n* = 6 per group; *p < 0.05, ***p < 0.001). One‐way ANOVA with Tukey's post hoc test; data are presented as mean ± SEM.
**Figure S6:** Evidence of Spp1 for improving synaptic function in the ischemic penumbra of the MCAO/R mouse model. (A–J) The mRNA levels of Vamp1, Snap25, Slc17a7 (A–C), Rims1, Rimbp2, Erc1 (D–F), Cask, Unc13b, Bsn, and Pclo (G–J) in the ischemic penumbra after shSpp1 was quantify by RT‐qPCR (*n* = 6–10 per group; *p < 0.05, **p < 0.01, ***p < 0.001). One‐way ANOVA with Tukey's post hoc test; data are presented as mean ± SEM.
**Figure S7:** Spp1 provides protection against synaptic dysfunction in OGD/R model. (A) RT‐qPCR was used to measure the mRNA expression levels of Syn1, Syp, Vamp2, Dlag4, Dlag2, and Homer1 in HT‐22 co‐cultured with Bend.3 and BV‐2 (*n* = 6 per group; *p < 0.05, **p < 0.01, ***p < 0.001). (B, C) Gray‐scale band image (B). Expression levels of Synapsin1, Synaptophysin, VAMP2, PSD93, PSD95, and Homer1 quantified using Western blot (*n* = 3 per group; *p < 0.05, **p < 0.01). (C) One‐way ANOVA with Tukey's post hoc test; data are presented as mean ± SEM.
**Figure S8:** Evidence of Spp1 for improving synaptic function in OGD/R model. (A–J) The mRNA levels of Vamp1, Snap25, Slc17a7 (A‐C), Rims1, Rimbp2, Erc1 (D–F), Cask, Unc13b, Bsn, and Pclo (G–J) in HT‐22 co‐cultured with Bend.3 and BV‐2 were quantified by RT‐qPCR (*n* = 6–10 per group; *p < 0.05, **p < 0.01, ***p < 0.001). One‐way ANOVA with Tukey's post hoc test; data are presented as mean ± SEM.

## Data Availability

The data that support the findings of this study are available from the corresponding author upon reasonable request.
