# Supplementary material for: Perivascular SPP1 Drives Microglial Synaptic Engulfment After Ischemic Stroke
Source: CNS Neurosci Ther. 2026 Apr 28;32(4):e70886. doi: 10.1002/cns.70886 (PMC13122273; doi:10.1002/cns.70886)
Supplement: Supplementary file 1 — Table S1: Primers used for Spp1 knockout via CRISPR/Cas9 gene editing. Table S2: Primers used for qPCR. Table S3: Antibody used for western blot. Figure S1: Evidence of synaptic deficits in the ischemic penumbra of the MCAO/R mouse model. (A–C) RT‐qPCR was used to quantify mRNA levels of presynaptic, postsynaptic, and active‐zone‐related genes in the ischemic penumbra of the Sham and MCAO/R groups (n = 6–10 per group; **p < 0.01, ***p < 0.001). Independent samples T‐test; data are presented as mean ± SEM. Figure S2: (A) Venn diagrams. (B) Heatmap of differentially expressed proteins in the Sham and MCAO/R groups. (C) Gene Ontology Enrichment of Spp1. Figure S3: Verification of Spp1−/− in Bend.3 of OGD/R model. (A) CRISPR/Cas9 gene editing technology was used to induce Spp1−/− in vitro. The expression level of Spp1 was quantified using RT‐qPCR in different groups (n = 6 per group; ***p < 0.001). (B, C) Western blot was used to quantify SPP1 protein level (n = 3 per group; *p < 0.05). (D) Representative immunofluorescence image of SPP1. Scale bar, 20 μm. (E) The ratio of SPP1 fluorescence intensity to the nucleus (n = 3 per group; ***p < 0.001). One‐way ANOVA with Tukey's post hoc test; data are presented as mean ± SEM. Figure S4: Bend.3 Spp1−/− modulate neuroinflammation in OGD/R model. (A–D) Microplate reader results showing IL‐1β (A), TNF‐α (B), IL‐10 (C), and TNF‐β (D) activities in the culture medium of Bend.3 co‐cultured with BV‐2 and HT‐22 (n = 6 per group; *p < 0.05, ***p < 0.001). One‐way ANOVA with Tukey's post hoc test; data are presented as mean ± SEM. Figure S6: Evidence of Spp1 for improving synaptic function in the ischemic penumbra of the MCAO/R mouse model. (A–J) The mRNA levels of Vamp1, Snap25, Slc17a7 (A–C), Rims1, Rimbp2, Erc1 (D–F), Cask, Unc13b, Bsn, and Pclo (G–J) in the ischemic penumbra after shSpp1 was quantify by RT‐qPCR (n = 6–10 per group; *p < 0.05, **p < 0.01, ***p < 0.001). One‐way ANOVA with Tukey's post hoc test; data are presented a [file CNS-32-e70886-s001.docx]

Perivascular SPP1 Drives Microglial Synaptic Engulfment After Ischemic Stroke

Chenchen Xu^1,2^, Xiaoxiao Li^1^, Nan Cheng^1,2^, Rui Zhao^1^, Xin Wang^1^, Wenxin Xia^1^, Jianjian Dong^1,2^*, Yongsheng Han^1,2,3^*

^1^Institute of Neurology, Anhui University of Chinese Medicine, Hefei, Anhui, 230012, China

^2^The Affiliated Hospital of Institute of Neurology, Anhui University of Chinese Medicine, Hefei, Anhui 230012, China

^3^Wannan Medical College, Wuhu, Anhui 241002, China

*Correspondence: Yongsheng Han ([hyysp@ahtcm.edu.cn](mailto:hyysp@ahtcm.edu.cn)); Jianjian Dong ([jjdong@ahtcm.edu.cn](mailto:jjdong@ahtcm.edu.cn))

**Supplementary Table S1** Primers used for *Spp1* knockout via CRISPR/Cas9 gene editing

| Genes |  | Primer sequences |
| --- | --- | --- |
| *Spp1* |  | gRNA-A1: AATGTTCGCCAGCGTCGCAA  AGG  gRNA-A2: CAGAGGAACACAGATTACCC  GGG |

**Supplementary Table S2** Primers used for qPCR

| Genes | Forward primer | Reverse primer |
| --- | --- | --- |
| *β-actin* | GTGACGTTGACATCCGTAAAGA | GTAACAGTCCGCCTAGAAGCAC |
| *Syn1* | *GATGCTAAATATGATGTGCGTGT* | *AATGTGATCCCTTCCGTCCTT* |
| *Syp* | *AGTGGGTCTTTGCCATCTTCG* | *CCGAGGAGGAGTAGTCACCAAC* |
| *Vamp2* | *GCTGGATGACCGTGCAGAT* | *GATGGCGCAGATCACTCCC* |
| *Dlag2* | *CGAACCAATCAGAAACGCTCC* | *TTCTTTCCACCCTCCGCTTG* |
| *Dlag4* | *GCAGGTTGCAGATCGGAGAC* | *ACTGATCTCATTGTCCAGGTGCT* |
| *Home1* | CCCTCTCTCATGCTAGTTCAGC | GCACAGCGTTTGCTTGACT |
| *Vamp1* | CATGCGTGTGAATGTGGACAA | GATGGCACAGATAGCTCCCAG |
| *Snap25* | ATCCGCAGGGTAACAAATGATG | CGGAGGTTTCCGATGATGC |
| *Slc17a7* | GGTGGAGGGGGTCACATAC | AGATCCCGAAGCTGCCATAGA |
| *Pclo* | CAGCCCTACCCGAACAAGC | CCGTTGTCACTTGAGCCTG |
| *Rims1* | CAAACCCTAGCCACCCCAG | CAGGTGTAGATTTGGAGCCAG |
| *Rimbp2* | CAGACCTGTCGGACATCATGG | AATCTTGAGGGCGTTGTGAGA |
| *Unc13b* | TTAAAAGAGCCAAATTCCAGGGT | TCCAGACGACTGATCTCAAACA |
| *Erc1* | AATGGGGCTTTGAGTAGTGAGG | AGGTATGGACTGGATTCTACACA |
| *Cask* | TGAAGAAGTAGTCAAACTGCCAG | TTTGTCCCGTACATTGCATCC |
| *Bsn* | GGGCAGCCAGAGAACAACTT | GGGACAGAGTAGGGTGACG |
| *Il6* | CCGGAGAGGAGACTTCACAGA | AGAATTGCCATTGCACAACTCTT |
| *Il1b* | ACCCTGCAGCTGGAGAGTGT | TTGACTTCTATCTTGTTGAAGACAAACC |
| *Il18* | AGACCTGGAATCAGACAACTTTGG | GGGTCACAGCCAGTCCTCTT |
| *Tnf* | GTAGCCCACGTCGTAGCAAAC | AGTTGGTTGTCTTTGAGATCCATG |
| *Ccl2* | TGTCTCAGCCAGATGCAGTTAAT | CCGACTCATTGGGATCATCTT |
| *Nos2* | CTATCTCCATTCTACTACTACCAGATCA | CCTGGGCCTCAGCTTCTCAT |
| *Cd68*  *Tjp1* | CACTTCGGGCCATGTTTCTC  GCCGCTAAGAGCACAGCAA | AGGACCAGGCCAATGATGAG  GCCCTCCTTTTAACACATCAGA |
| *Vcam1* | TTGGGAGCCTCAACGGTACT | GCAATCGTTTTGTATTCAGGGGA |
| *Mmp9* | GCAGAGGCATACTTGTACCG | TGATGTTATGATGGTCCCACTTG |
| *Ocln* | TGAAAGTCCACCTCCTTACAGA | CCGGATAAAAAGAGTACGCTGG |
| *Cldn5* | GCAAGGTGTATGAATCTGTGCT | GTCAAGGTAACAAAGAGTGCCA |
|  |  |  |

**Supplementary Table S3** Antibody used for western blot

| Anti-body | Cat | Brand |
| --- | --- | --- |
| Synapsin1 | 5297 | Cell Signaling Technology |
| Synaptophysin | 36406 | Cell Signaling Technology |
| PSD93 | 19046 | Cell Signaling Technology |
| PSD95 | 3450 | Cell Signaling Technology |
| VAMP2 | GB11451 | Service bio |
| SPP1 | 22952-1-AP | Proteintech |
| C3 | A26625PM | ABclonal |
| ZO-1 | 511417 | Zenbio |
| VCAM-1 | R012515 | Eyizyme |
| MMP9 | GB11132 | Service bio |
| Claudin5 | 3432141 | Zenbio |
| Occludin | 502601 | Zenbio |
| α-Tubulin | LF213S | Epizyme |
| *β*-Tubulin | GB11017 | Service bio |
| *β*-actin | GB11001 | Service bio |
| NeuN | GB11138 | Service bio |
| GAP43 | GB11095 | Service bio |
| VEGFA | GB11034B | Service bio |


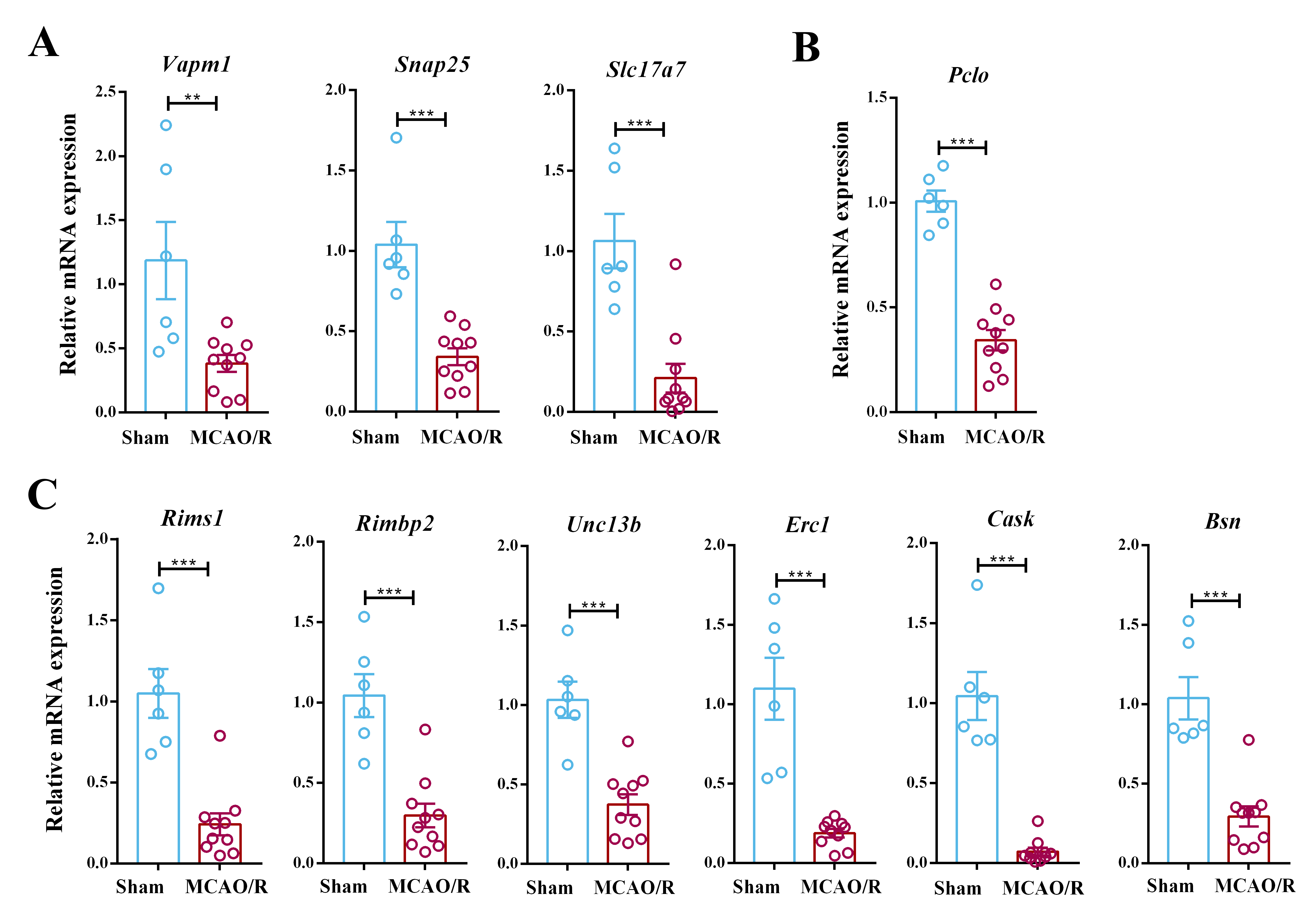
 **Supplementary Fig. S1 Evidence of synaptic deficits in the ischemic penumbra of the MCAO/R mouse model. A-C** RT-qPCR was used to quantify mRNA levels of presynaptic, postsynaptic, and active-zone-related genes in the ischemic penumbra of the Sham and MCAO/R groups. (*n* = 6–10 per group; ***P* < 0.01, ****P* < 0.001). Independent Samples T-test; Data are presented as mean ± SEM.


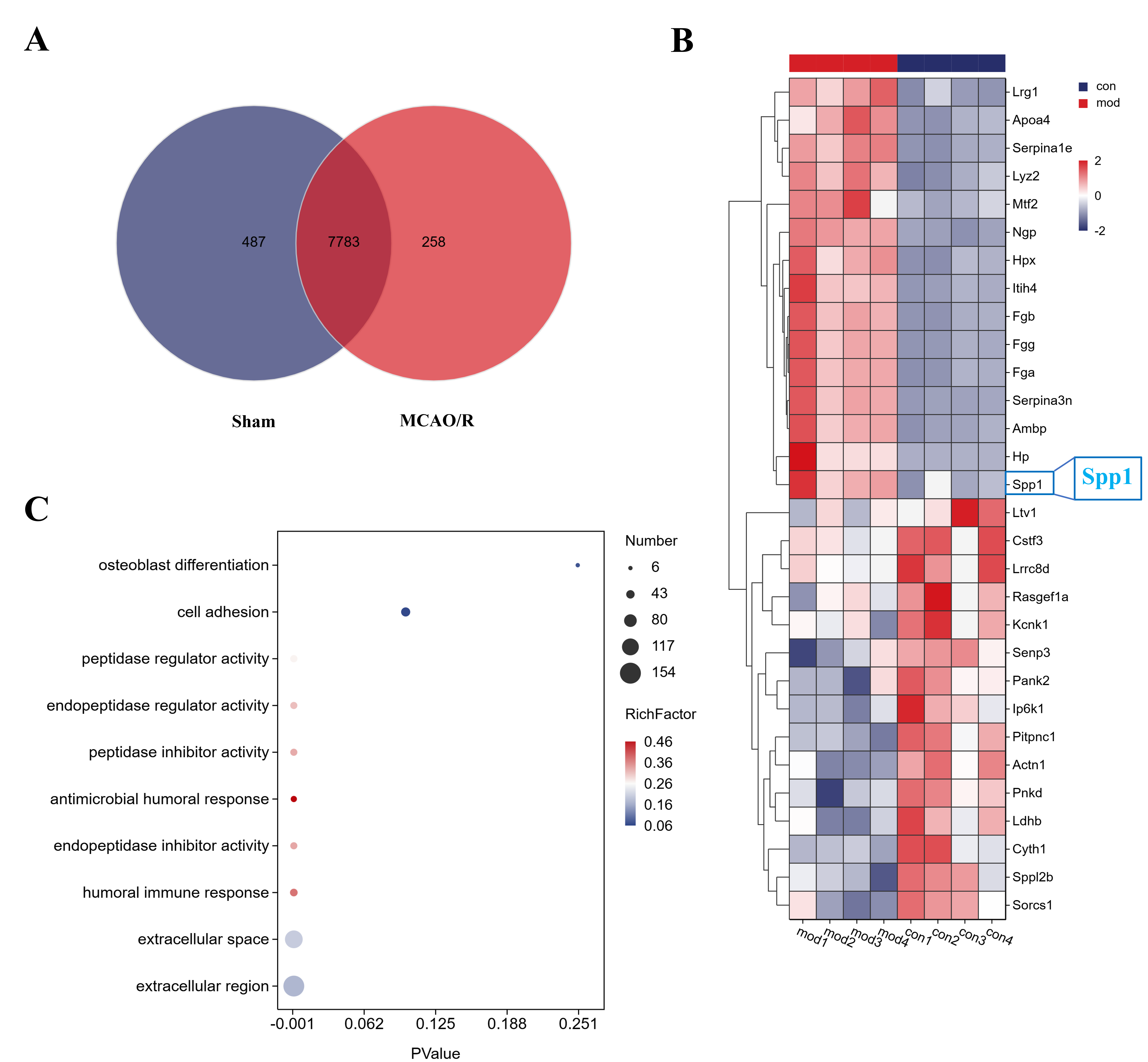


**Supplementary Fig. S2 A** Venn diagrams. **B** Heatmap of differentially expressed proteins in the Sham and MCAO/R groups. **C** Gene Ontology Enrichment of *Spp1.*


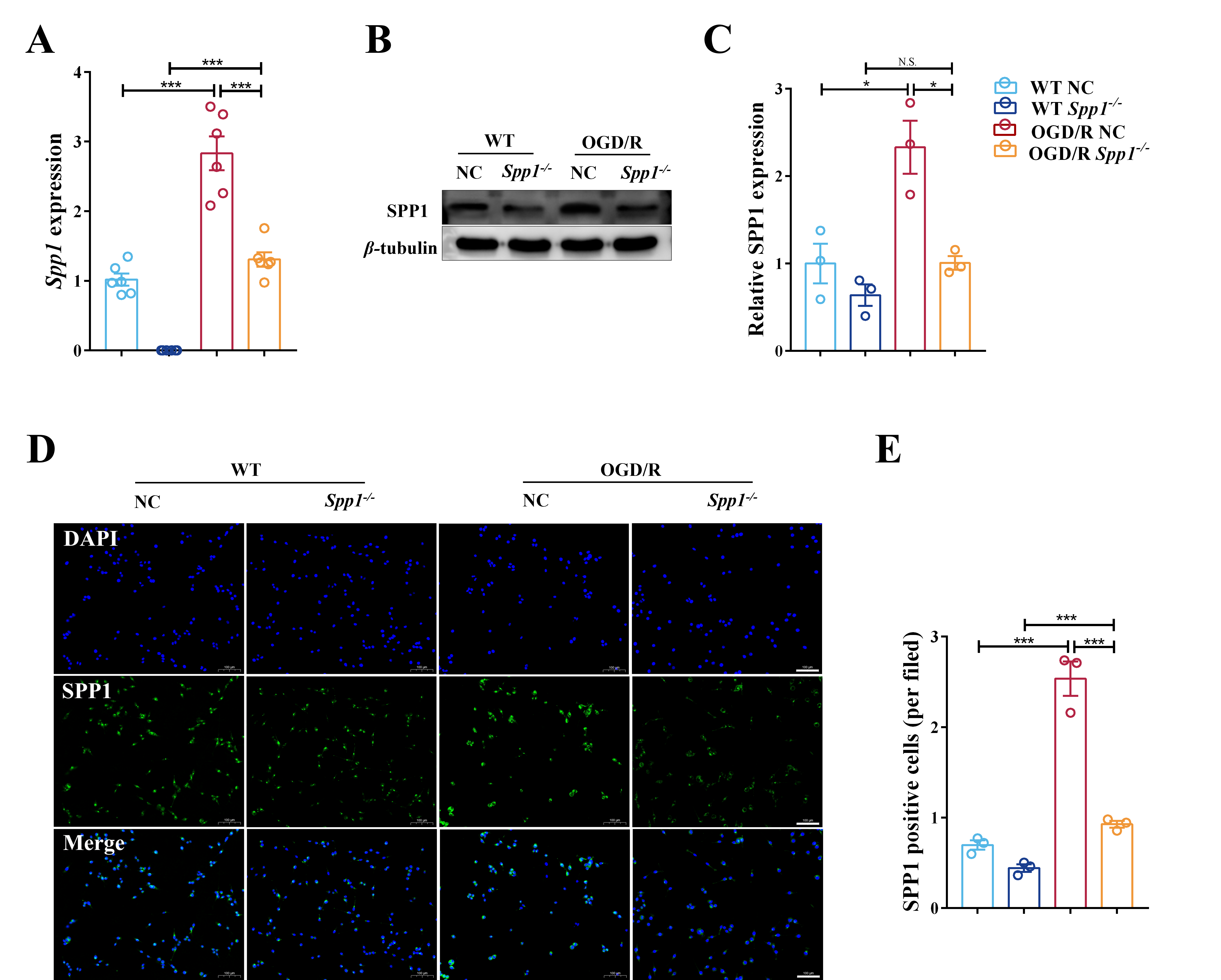


**Supplementary Fig. S3 Verification of *Spp1^-/-^* in Bend.3 of OGD/R model.** **A** CRISPR/Cas9 gene editing technology was used to induce *Spp1^-/-^* in vitro. The expression level of *Spp1* was quantified using RT-qPCR in different groups (*n* = 6 per group; ****P* < 0.001). **B, C** Western blot was used to quantify SPP1 protein level (*n* = 3 per group; **P* < 0.05,). **D** Representative immunofluorescence image of SPP1. Scale bar, 20 µm. **E** The ratio of SPP1 fluorescence intensity to the nucleus. (*n* = 3 per group; ****P* < 0.001). One-way ANOVA with Tukey’s post-hoc test; Data are presented as mean ± SEM.


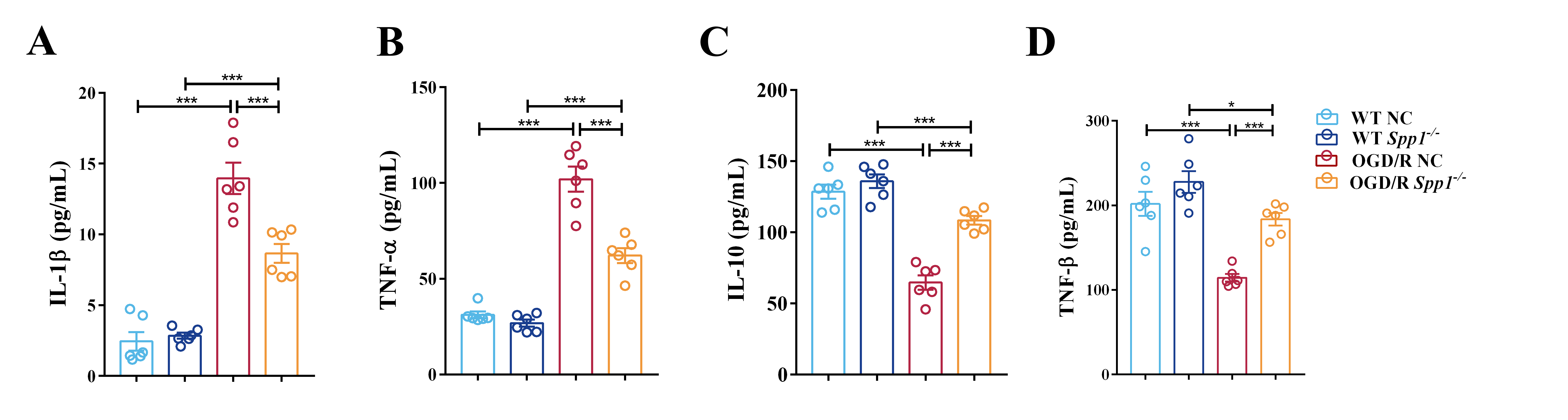


**Supplementary Fig. S4 Bend.3 *Spp1^-/-^* modulate neuroinflammation in OGD/R model. A-D** Microplate reader results showing IL-1β (**A**), TNF-α (**B**), IL-10 (**C**), and TNF-β (**D**) activities in the culture medium of Bend.3 co-cultured with BV-2 and HT-22 (*n*= 6 per group; **P* < 0.05, ****P* < 0.001). One-way ANOVA with Tukey’s post-hoc test; Data are presented as mean ± SEM.


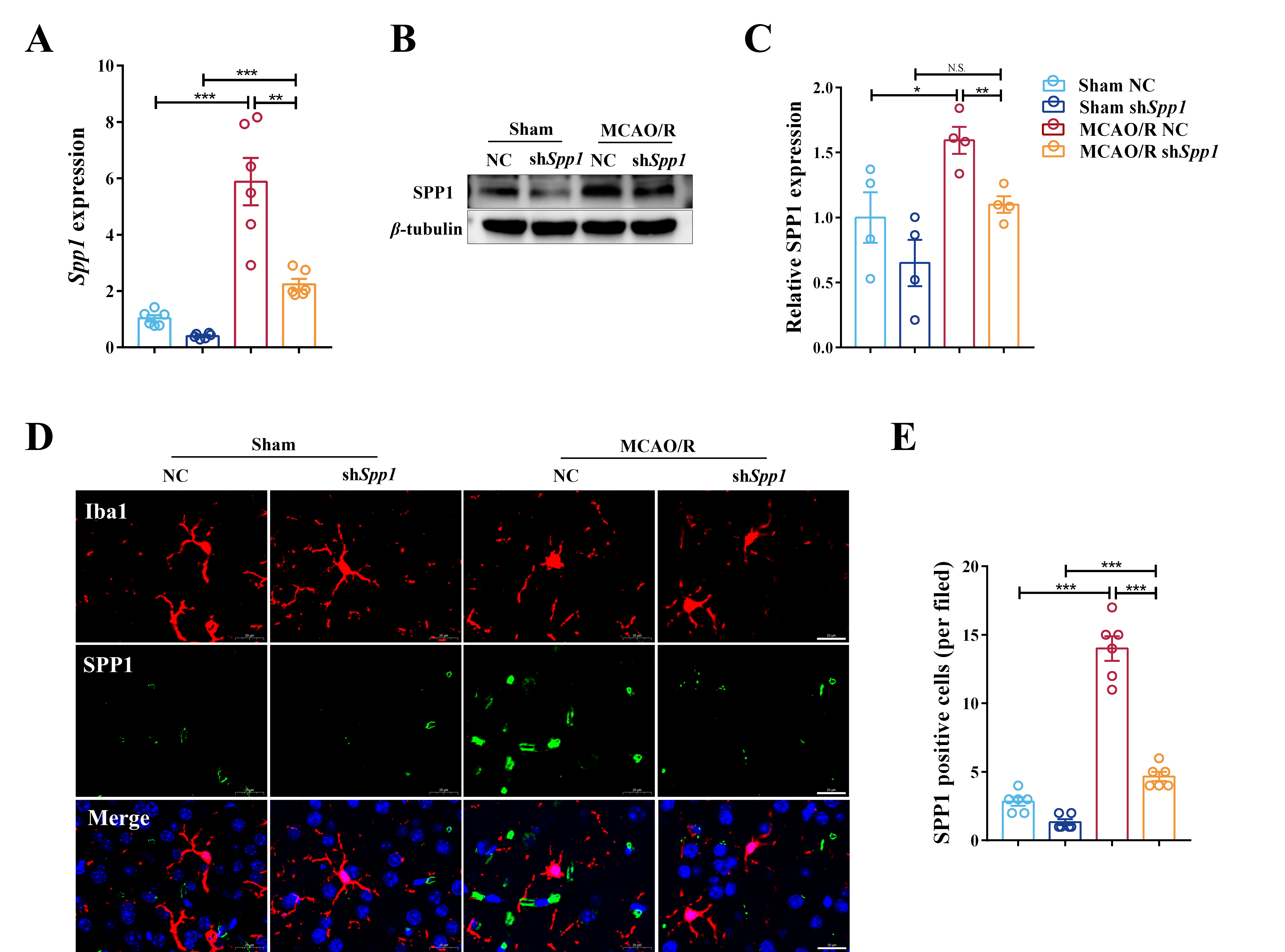
 **Supplementary Fig. S5 Verification of sh*Spp1* treatment in the ischemic penumbra of the MCAO/R mouse model. A** Quantification of mRNA expression levels of *Spp1* using RT-qPCR in the ischemic penumbra of different groups (*n* = 6 per group; ***P* < 0.01, ****P* < 0.001). **B, C** Western blot was used to quantify SPP1 protein level. (*n* = 4 per group; **P* < 0.05, ***P* < 0.01). **D** Representative immunofluorescence double-staining images for Iba1/SPP1. Scale bar, 20 µm. **E** SPP1 positive cells per field the ischemic penumbra of different groups after sh*Spp1*. (*n* = 6 per group; ****P* < 0.001). One-way ANOVA with Tukey’s post-hoc test; Data are presented as mean ± SEM.


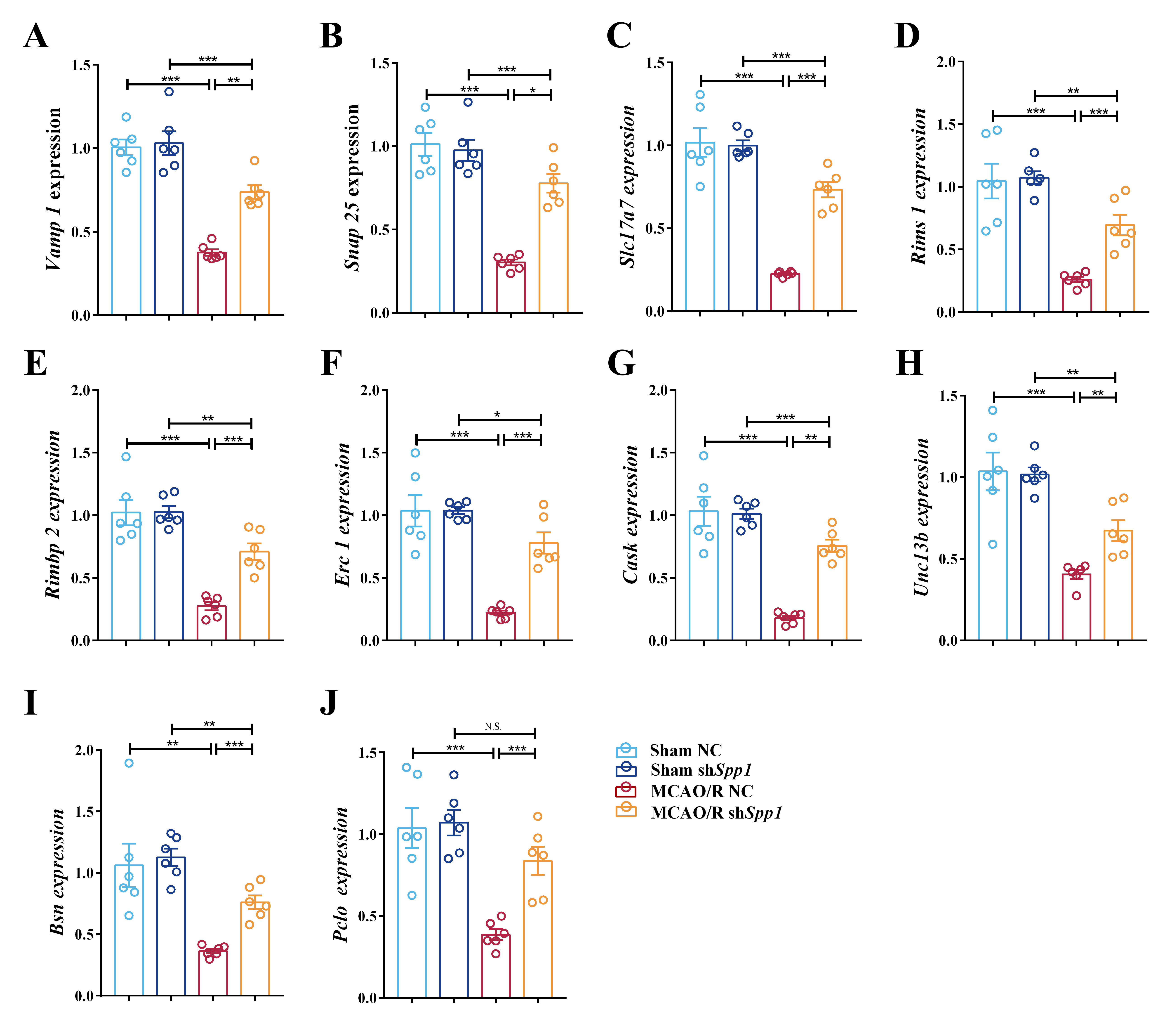


**Supplementary Fig. S6 Evidence of *Spp1* for improving synaptic function in the ischemic penumbra of the MCAO/R mouse model. A-J** The mRNA levels of *Vamp1, Snap25, Slc17a7* (**A-C**), *Rims1, Rimbp2, Erc1* (**D-F**), *Cask, Unc13b, Bsn, and Pclo* (**G-J**) in the ischemic penumbra after sh*Spp1* was quantify by RT-qPCR. (*n* = 6–10 per group; **P* < 0.05, ***P* < 0.01, ****P* < 0.001). One-way ANOVA with Tukey’s post-hoc test; Data are presented as mean ± SEM.


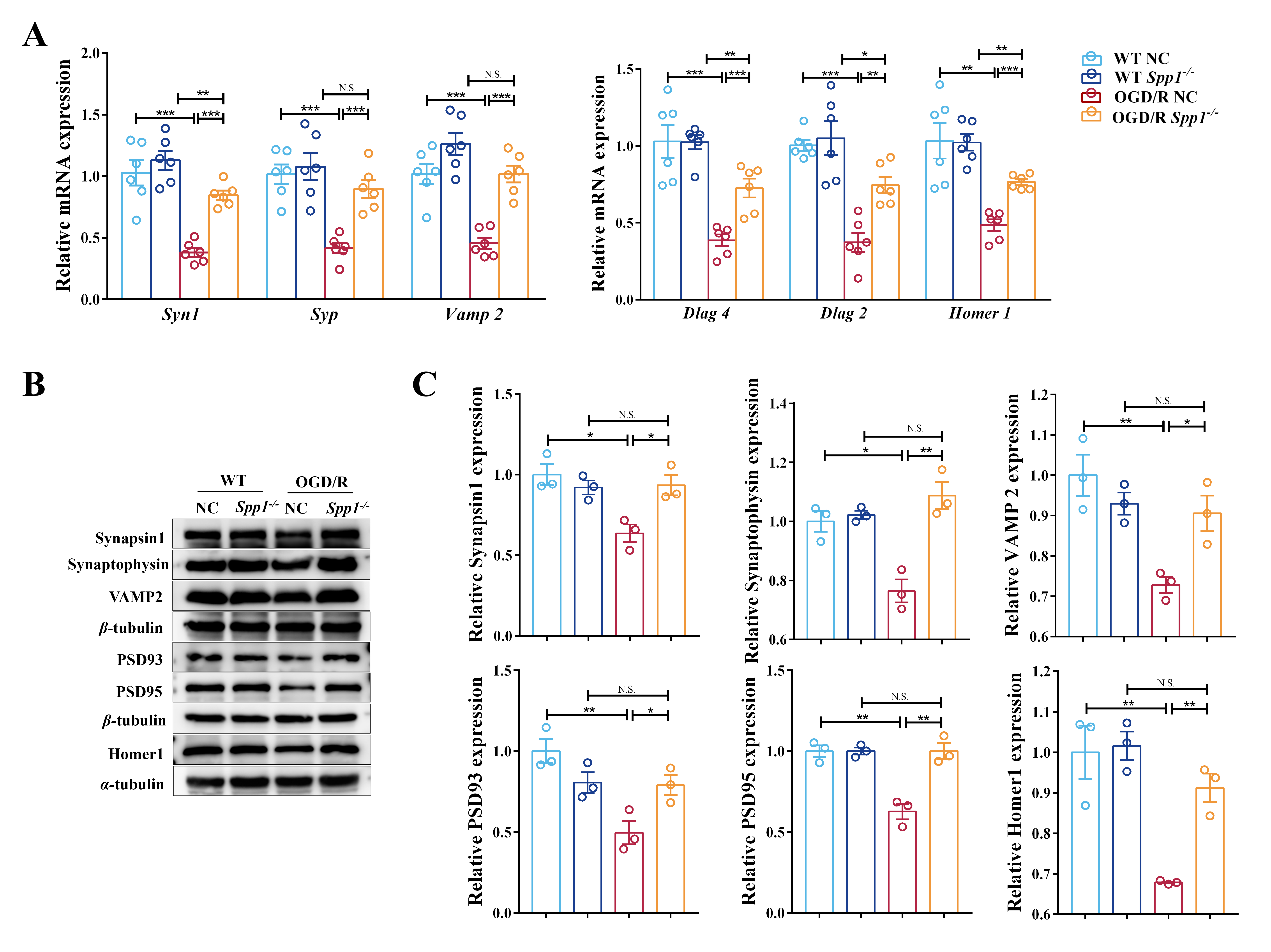


**Supplementary Fig. S7 *Spp1* provides protection against synaptic dysfunction in OGD/R model. A** RT-qPCR was used to measure the mRNA expression levels of *Syn1*, *Syp*, *Vamp2*, *Dlag4*, *Dlag2*, and *Homer1* in HT-22 co-cultured with Bend.3 and BV-2 (*n* = 6 per group; **P* < 0.05, ***P* < 0.01, ****P* < 0.001). **B, C** Gray-scale band image **(B)**. Expression levels of Synapsin1, Synaptophysin, VAMP2, PSD93, PSD95, and Homer1 quantified using Western blot (*n* = 3 per group; **P* < 0.05, ***P* < 0.01) **(C)**. One-way ANOVA with Tukey’s post-hoc test; Data are presented as mean ± SEM.


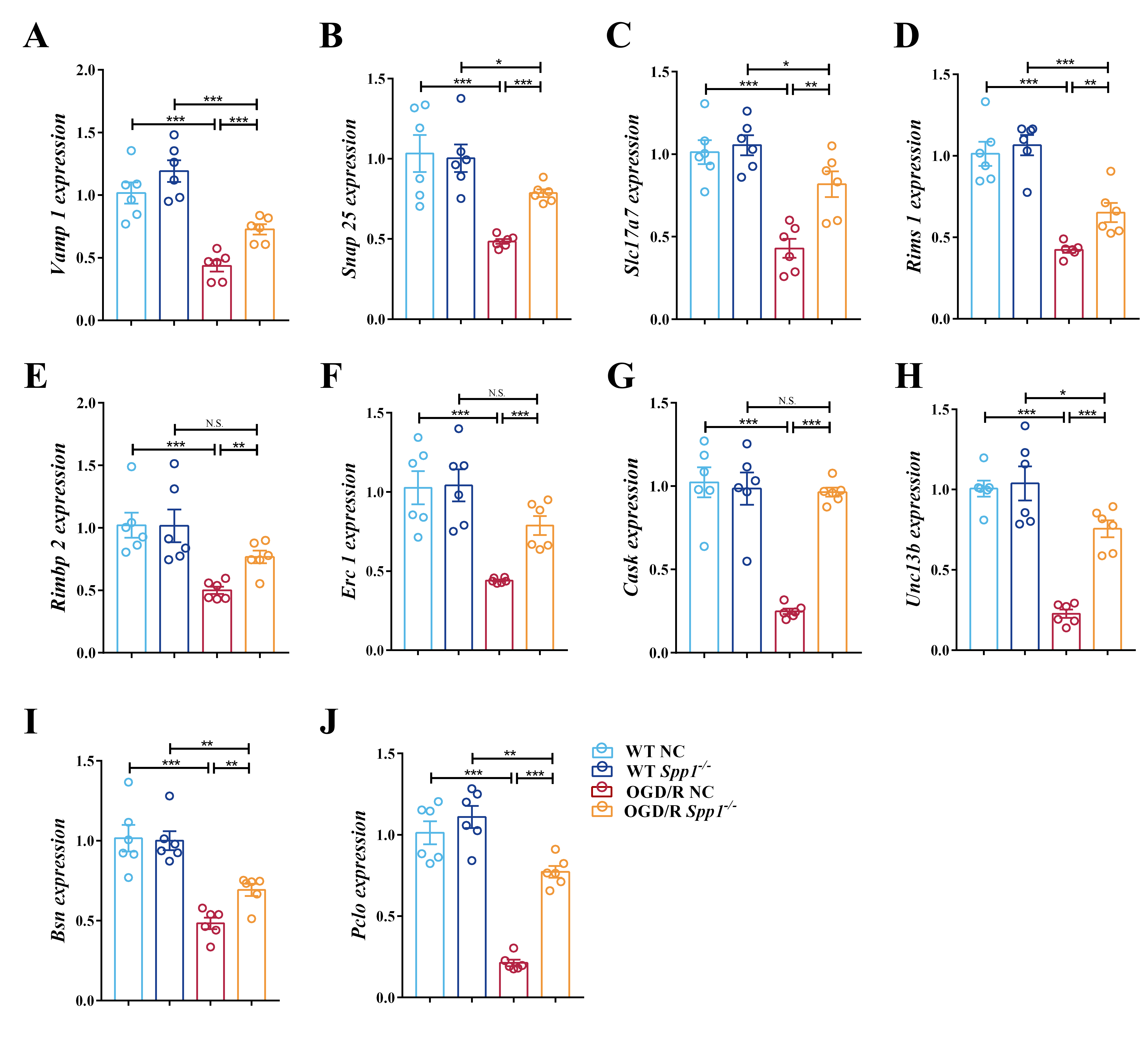


**Supplementary Fig. S8 Evidence of *Spp1* for improving synaptic function in OGD/R model. A-J** The mRNA levels of *Vamp1, Snap25, Slc17a7* (**A-C**), *Rims1, Rimbp2, Erc1* (**D-F**), *Cask, Unc13b, Bsn, and Pclo* (**G-J**) in HT-22 co-cultured with Bend.3 and BV-2 were quantified by RT-qPCR. (*n* = 6–10 per group; **P* < 0.05, ***P* < 0.01, ****P* < 0.001). One-way ANOVA with Tukey’s post-hoc test; Data are presented as mean ± SEM.
